# Dysfunction of infiltrating cytotoxic CD8^+^ T cells within the graft promotes murine kidney allotransplant tolerance

**DOI:** 10.1172/JCI179709

**Published:** 2024-06-18

**Authors:** Takahiro Yokose, Edward S. Szuter, Ivy Rosales, Michael T. Guinn, Andrew S. Liss, Taisuke Baba, David A. Ruddy, Michelle Piquet, Jamil Azzi, A. Benedict Cosimi, Paul S. Russell, Joren C. Madsen, Robert B. Colvin, Alessandro Alessandrini

**Affiliations:** 1 Center for Transplantation Sciences, Department of Surgery and; 2 Department of Surgery, Massachusetts General Hospital and Harvard Medical School, Boston, Massachusetts, USA.; 3 Department of Pathology, Massachusetts General Hospital, Boston, Massachusetts, USA.; 4 Michael E. DeBakey Department of Surgery, Baylor College of Medicine, Houston, Texas, USA.; 5 Novartis Biomedical Research, Oncology, Cambridge, Massachusetts, USA.; 6 Transplantation Research Center, Brigham and Women’s Hospital and Harvard Medical School, Boston, Massachusetts, USA.; 7 Division of Cardiac Surgery, Massachusetts General Hospital, Boston, Massachusetts, USA.

**Keywords:** Immunology, Transplantation, Organ transplantation, T cells, Tolerance

## Abstract

Tolerance of mouse kidney allografts arises in grafts that develop regulatory tertiary lymphoid organs (rTLOs). Single-cell RNA-seq (scRNA-seq) data and adoptive transfer of alloreactive T cells after transplantation showed that cytotoxic CD8^+^ T cells are reprogrammed within the accepted graft to an exhausted/regulatory-like phenotype mediated by IFN-γ. Establishment of rTLOs was required because adoptive transfer of alloreactive T cells prior to transplantation results in kidney allograft rejection. Despite the presence of intragraft CD8^+^ cells with a regulatory phenotype, they were not essential for the induction and maintenance of kidney allograft tolerance since renal allotransplantation into CD8-KO recipients resulted in acceptance and not rejection. Analysis of scRNA-seq data from allograft kidneys and malignant tumors identified similar regulatory-like cell types within the T cell clusters and trajectory analysis showed that cytotoxic CD8^+^ T cells are reprogrammed into an exhausted/regulatory-like phenotype intratumorally. Induction of cytotoxic CD8^+^ T cell dysfunction of infiltrating cells appears to be a beneficial mechanistic pathway that protects the kidney allotransplant from rejection through a process we call “defensive tolerance.” This pathway has implications for our understanding of allotransplant tolerance and tumor resistance to host immunity.

## Abstract 2

scRNA-Seq and adoptive cell transfer reveal graft-infiltrating cytotoxic CD8^+^ T cells are reprogrammed to an exhausted/regulatory phenotype within accepted kidney allografts in mice.

## Graphical abstract

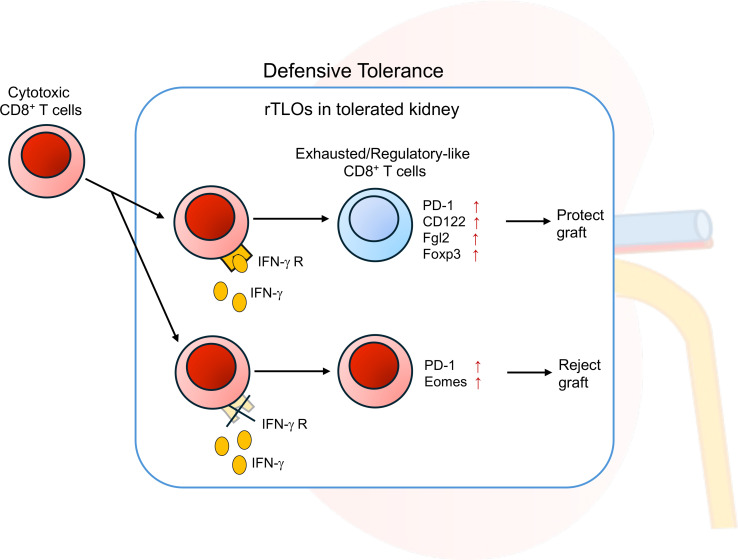

## Introduction

Traditionally, inflammatory and regulatory immune responses after transplantation were thought to be primarily generated in secondary lymphoid organs (1, 2). However, it has been increasingly recognized that both deleterious and beneficial alloimmune responses can also be regulated locally within the graft (3). Our laboratory has focused on understanding how murine kidney allografts are spontaneously accepted across fully mismatched donor-recipient strain combinations (e.g., DBA/2J to C57BL/6 [B6]) without any immunosuppressive drug treatment and maintained without the development of acute and chronic rejection in all cases.

We have shown that DBA/2J-to-B6 spontaneously accepted kidney allografts develop localized, intragraft aggregates of various lymphocytes around the small vessels of the graft and represent novel regulatory tertiary lymphoid organs (rTLOs), similar to tertiary lymphoid structures (TLSs) found in solid tumors (4, 5). These structures have been shown to be consistently present in accepted kidney allografts and absent in rejected allografts. They contain Foxp3^+^ regulatory T cells (Tregs) and have hence been named Treg-rich organized lymphoid structures (TOLS) (4). Depletion of Tregs prior to 6 months from recipients results in dissolution of these rTLOs and acute rejection of the graft (4); however, Treg depletion at 24 weeks does not result in rejection (6). We have previously reported that spontaneously accepted DBA/2J kidneys in B6 can induce systemic, donor-specific tolerance of cardiac allografts and is dependent on Foxp3^+^ cells (7). We further showed that Foxp3 induction by plasmacytoid dendritic cells in vitro closely correlated with strain combinations that led to kidney allograft acceptance (8). While Tregs are typically CD4^+^CD25^+^Foxp3^+^, CD8^+^ Tregs have also been shown to play a role in tolerance. These cells are characterized by CD8^+^FGL2^+^CD122^+^ markers and have been found to induce regulatory B cells and inhibit maturation of dendritic cells through interactions of FGL2 and its receptor FcγRIIB (9–12). Interestingly, upon analysis of single-cell RNA-seq (scRNA-seq) data from pancreatic and colorectal tumors in mice, we have identified similar cells within the CD8^+^ T cell cluster that express *Fgl2* and *Il2rb* (CD122).

We initially observed that both kidney allografts that were ultimately rejected and those that were accepted had similar levels of leukocyte infiltration 1 week after transplantation (5). Importantly, however, bulk mRNA analysis and digital spatial protein profiling analyses identified differential gene expression in the allografts that went on to rejection versus acceptance. The most notable difference was the increase in transcripts and proteins related to Treg phenotype and elevated function in accepted kidney allografts (5). Furthermore, bulk mRNA analysis identified early infiltration of cytotoxic CD8^+^ T cells in grafts destined for acceptance, which diminished with time (5). Digital spatial protein profiling of rTLOs within accepted kidneys showed that markers of exhaustion (i.e., PD-1, LAG3, and TIM3) are highly expressed when compared with the adjacent cortex at 1–8 weeks after transplantation (5). While previous studies have shown that Foxp3^+^ cells in rTLOs play an important role in downregulating alloimmune responses, several important facets of how rTLOs promote tolerance and how infiltrating cytotoxic CD8^+^ T cells might be reprogrammed remain undefined. We hypothesized that rTLOs might disarm or reprogram CD8^+^ effector cells.

The T cell population consists of naive cells and memory cells that arise after sensitization to donor antigens. Memory cells can generate more effector cells than naive cells, and infiltration of memory cells usually triggers graft rejection (13). Thus, adoptive transfer of alloreactive CD8^+^ cells after kidney or heart transplantation causes graft rejection (14). However, recipients of spontaneously accepted kidney grafts showed increased blood urea nitrogen (BUN) levels after adoptive transfer of donor-sensitized splenocytes, but then recovered and maintained normal renal function (15). We hypothesized that cytotoxic CD8^+^ T cells infiltrating the allograft are reprogrammed to regulatory-like and/or exhausted CD8^+^ T cells within the kidney allograft.

To test this hypothesis, we analyzed scRNA-seq data from different time points after transplantation and have characterized the gene expression patterns of these cells. We found that the gene expression signature of infiltrating cytotoxic CD8^+^ T cells shifts from an effector phenotype to one that is reflective of exhaustion/regulation only within the accepted kidney allografts. Adoptive transfer of alloreactive T cells prior to transplantation resulted in rejection of the kidney. In contrast, adoptive transfer after transplantation did not lead to rejection. We also showed that transplantation of DBA/2J kidney allografts into CD8-KO recipients results in the spontaneous acceptance of the renal grafts. These observations emphasize that an ongoing state of tolerance is required for reprogramming of cytotoxic CD8^+^ T cells to non-effector cell types and that such reprogramming is necessary to protect the kidney from CD8^+^ T cell cytotoxicity, but is not required for the induction and maintenance of kidney allograft acceptance. This intragraft reprogramming appears to be a secondary, regulatory, or protective mechanism. Overall, these findings help to clarify not only how allotransplant tolerance in this model is maintained, but also appear to have implications in our understanding of how tumors escape the host’s immune system.

## Results

### scRNA-seq and flow cytometry show that CD8^+^ T cells constitute the significant population of infiltrating immune cells in the early stages of kidney allograft acceptance.

Bulk mRNA transcripts from accepted DBA/2J kidney allografts transplanted into B6 recipients revealed that the CD8^+^ T cells decreased with time (5). Furthermore, digital spatial protein profiling of rTLOs within accepted kidney grafts showed that markers of exhaustion (PD-1, LAG3, TIM3) are highly expressed within rTLOs when compared with the adjacent cortex of the accepted kidney allograft at 1–8 weeks after transplantation (5). The rTLOs begin to form 1 week after transplantation and are fully formed at around 2 to 3 weeks (5, 7). Tregs play an important role in the induction and maintenance of tolerance in the early phase following transplantation and as mentioned, alternative tolerance mechanisms are at play at 24 weeks after transplantation since Treg depletion does not result in rejection at this time (6).

**Figure 1:**
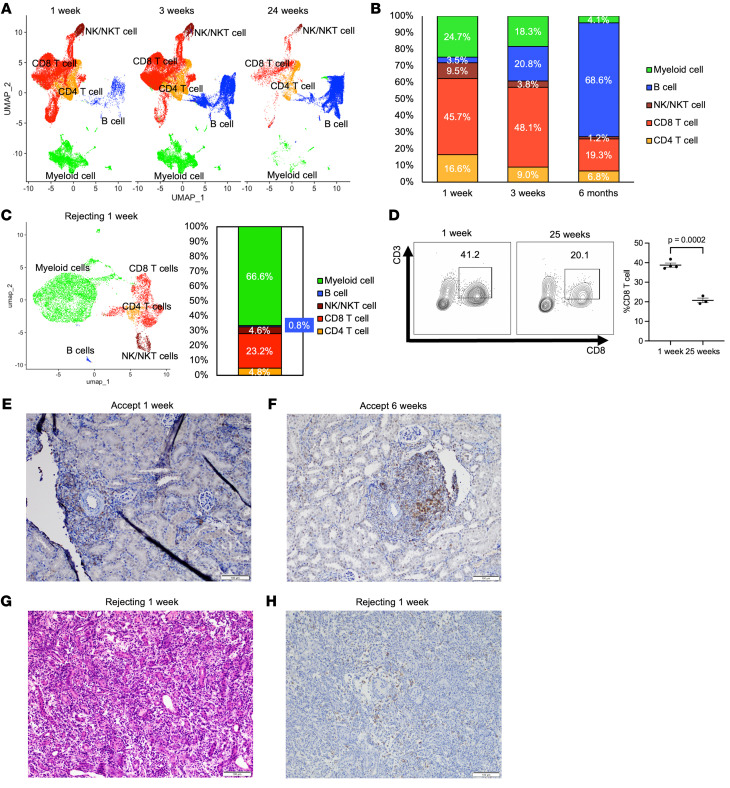
scRNA-seq and flow cytometry show that CD8^+^ T cells constitute the significant population of infiltrating immune cells in the early stages of kidney allograft acceptance. (**A–C**) UMAP plots (**A** and **C**) and bar graphs (**B** and **C**) of immune cell populations at each time point in scRNA-seq data of accepted kidney allografts (**A** and **B**) and rejecting kidney allografts at 1 week after transplantation (**C**). (**D**) Flow cytometric analysis of CD8^+^ T cells in accepted kidney allografts at 1 week and 25 weeks after transplantation. Data are represented as mean ± SEM, compared by 2-tailed Student’s *t* test. (**E–H**) Histopathological findings in accepted kidney allografts at 1 week (**E**) and 6 weeks (**F**) after transplantation and rejecting kidney allografts at 1 week (**G** and **H**) after transplantation. (**E**, **F**, and **H**) Immunohistochemistry of CD8 staining. (**G**) H&E staining. Scale bars: 100 μm.

We performed scRNA-seq analysis to resolve the gene expression evolution of immune cell subtypes within the accepted kidney 1, 3, and 24 weeks after transplantation. scRNA-seq analyses of CD45^+^ cells isolated from accepted kidney allografts revealed that the major immune cell population at 1 week and 3 weeks consists of CD8^+^ T cells. However, by 24 weeks, the CD8^+^ T cell population decreased from 48% to 19% of CD45^+^ cells, at which point the B cell population became the dominant population (Figure 1, A and B). Uniform manifold approximation and projection (UMAP) plots of integrated data for each time point corroborate these changes with a shift from a T cell–rich to a B cell–rich environment (Figure 1, A and B). In contrast, in the recipient’s spleen, as well as naive spleen, the B cell population was consistently the major cell population during the same time period, with CD8^+^ T cells representing the next most abundant population (Supplemental Figure 1, A–C; supplemental material available online with this article; https://doi.org/10.1172/JCI179709DS1).

For comparison, we analyzed scRNA-seq data of isolated CD45^+^ cells isolated from rejecting kidney allografts at 1 week after transplantation using an established kidney allograft rejection model (i.e., B6 to DBA/2J) that has a median survival time (MST) of 9 to 10 days (8). UMAP and bar plots of rejecting renal allografts showed that the myeloid cell cluster was the major population (67%), with CD8^+^ T cells being the second most abundant one (23%) (Figure 1C). Interestingly, principal component analysis (PCA) and sample-to-sample distance analysis in total immune cells obtained from kidneys and spleens from accepted, rejecting, and naive mice showed clustering of 1- and 3-week accepted kidney samples with 24-week recipient spleen samples and clustering of 24-week accepted kidney samples with naive, 1-, and 3-week recipient spleen samples; kidney and spleen samples from rejecting recipients showed the most variation with other samples (Supplemental Figure 1, D and E).

Flow cytometry of CD45^+^ cells isolated from DBA/2J kidneys transplanted into WT B6 at 1 and 25 weeks after transplantation showed that kidney allografts contain high levels of infiltrating CD8^+^ T cells. However, this number decreased with time (38.8% at 1 week and 20.7% at 25 weeks, *P* = 0.0002) (Figure 1D). The percentage of CD4^+^ T cells stayed relatively constant throughout the same time period (14% and 12%) (Supplemental Figure 1F), while B cells increased in percentage (7% at 1 week and 74% at 25 weeks) (6). In contrast, native B6 and DBA/2J kidneys had significantly fewer lymphocytes (9.7% and 9.3%, respectively) than accepted kidney allografts (55.0%) or recipient’s spleen (94.4%) at 1 week after transplant, with CD8^+^ T cells comprising only 1%–2% of total isolated lymphocytes (Figure 1D and Supplemental Figure 1G).

DBA/2J-to-B6 spontaneously accepted kidney allografts at 1 week after transplantation showed widespread cortical interstitial infiltrates of T cells with focal collections around interlobular and arcuate arteries, followed by progressive formation of periarterial rTLOs at 2–3 weeks after transplantation (Supplemental Figure 1H). Immunohistochemical analysis of rTLOs within kidney allografts between 1 and 6 weeks showed that they were rich in CD3^+^Foxp3^+^ cells (Supplemental Figure 1I) and CD8^+^ T cells (Figure 1, E and F). In contrast, rejecting kidney allografts (i.e., B6 to DBA/2J) (8) showed diffuse infiltration of lymphocytes without rTLO formation (Figure 1G). CD8^+^ cells infiltrate diffusely rather than perivascularly in rejected kidney allografts (Figure 1H).

### Intragraft gene expression within the CD8^+^ T cell clusters in accepted kidney allografts shows changes from a cytotoxic to an exhausted/regulatory-like phenotype.

**Figure 2:**
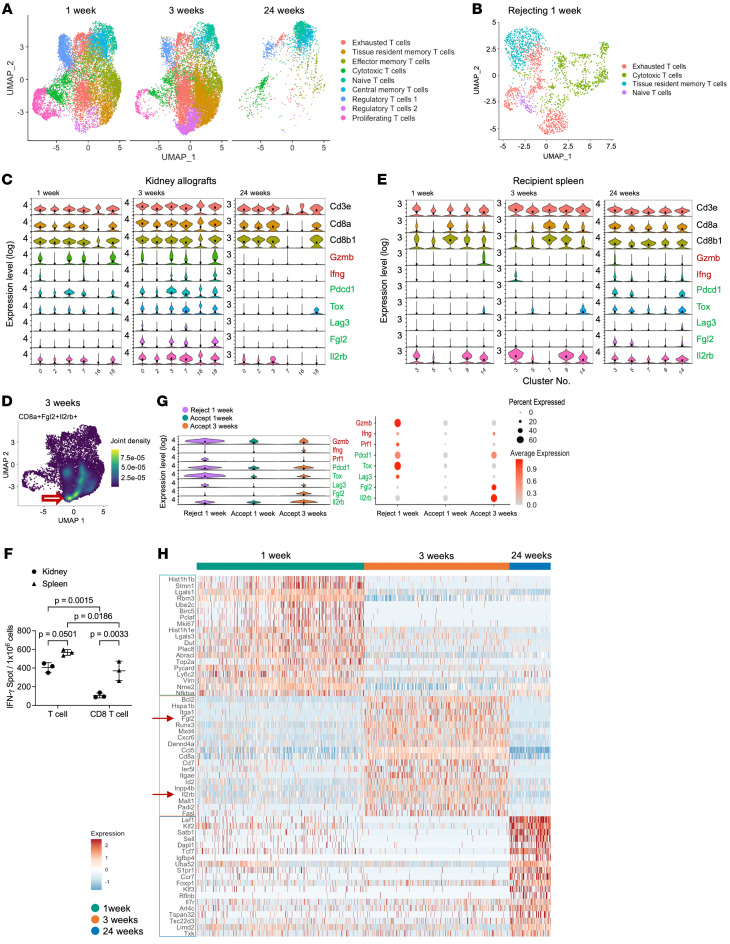
Intragraft gene expression within the CD8^+^ T cell clusters in accepted kidney allografts shows changes from a cytotoxic to an exhausted/regulatory-like phenotype. (**A** and **B**) UMAP plots of CD8^+^ T cell cluster subsets at each time point in accepted kidney allografts (**A**) and rejecting kidney allografts at 1 week (**B**). (**C** and **E**) Violin plots of cytotoxic (red) and exhaustion/regulatory (green) genes in CD8^+^ T cell clusters in accepted kidney allografts (**C**) and accepted recipient’s spleen (**E**). The vertical axis indicates log-ranked gene expression levels. Mean expression levels are indicated as black points. (**D**) Density plot of *CD8a*^+^*Fgl2*^+^*Il2rb*^+^ cells in accepted kidney allografts at 3 weeks after transplantation. (**F**) Bar graph shows ELISPOT analysis of IFN-γ production in T cells and CD8^+^ T cells obtained from accepted kidney allograft and recipient’s spleen at 24 weeks after transplantation. Data are represented as mean ± SD, compared by 2-way ANOVA test. (**G**) Violin plot and dot plot of cytotoxic (red) and exhaustion/regulatory (green) genes in CD8^+^ T cell clusters, comparing rejecting grafts at 1 week and accepted kidney allografts at 1 and 3 weeks. The black dot in violin plots indicates the mean value of expression levels. (**H**) Heatmap of top differentially expressed genes in CD8^+^ T cell clusters at each time point in accepted kidney allografts.

CD8^+^ T cell clusters changed over time, as shown by scRNAseq analysis (Figure 2A). UMAP plot analysis showed that cytotoxic and proliferative CD8^+^ T cells were the major subset at 1 week, but at 3 weeks, exhausted cells, regulatory-like cells, and tissue-resident memory CD8^+^ T cells became the major subset. There are 2 potentially CD8^+^ Treg subtypes in accepted renal allografts, CD8^+^Foxp3^+^ Tregs and CD8^+^IL-2RB^+^ Tregs (Figure 2A and Supplemental Figure 2, A and B) (16). At 24 weeks, central memory CD8^+^ T cells and naive cells became the major subset (Figure 2A and Supplemental Figure 2, A and B). CD8^+^ Treg subsets represented a small fraction in rejecting grafts compared with the accepted grafts, with cytotoxic T cells, exhausted T cells, and tissue-resident memory T cells making up most of the subsets and no Treg cell cluster (Figure 2B and Supplemental Figure 2, C and D).

Temporal scRNA-seq analysis within the CD8^+^ T cell clusters shows a shift from a cytotoxic to an exhausted/regulatory phenotype (Figure 2C). Violin plots of gene expression levels of cytotoxic and proinflammatory mediators (*Gzmb*, granzyme B; *Ifng*, IFN-γ) in CD8^+^ T cells appeared early and decreased with time, whereas exhaustion markers (*Pdcd1*, PD-1; *Lag3*, LAG3; *Tox*, TOX) and CD8^+^ Treg markers (*Fgl2*, FGL2; *Il2rb*, CD122) (10, 16) within the same clusters began to be expressed at 1 week, and peaked at 3 weeks before decreasing by 24 weeks (Figure 2C). The increased expression of *Fgl2* within the CD8^+^ T cell clusters at 3 weeks is particularly interesting. *Fgl2* encodes fibrinogen-like protein 2 (FGL2), which has been shown to be secreted by CD8^+^ Tregs and functions to induce regulatory B cells and inhibit dendritic cell maturation (9, 10). Density plots show that *Cd8a*^+^*Fgl2*^+^*Il2rb*^+^ cells were present in the CD8^+^ Treg cluster at 3 weeks (Figure 2D).

These patterns were not observed in CD8^+^ T cells from recipient splenocytes (Figure 2E). In contrast to intragraft cells, cytotoxic and proinflammatory mediators (*Gzmb* and *Ifng*) in CD8^+^ T cell clusters in recipient spleen cells were expressed only weakly at 1 and 3 weeks after transplantation, but increased at 24 weeks. Furthermore, exhaustion and regulatory markers (*Pdcd1*, *Lag3*, *Tox*, *Fgl2*, and *Il2rb*) were barely expressed at 1 and 3 weeks, but were upregulated at 24 weeks (Figure 2E). In naive spleen, cytotoxic, exhausted, and regulatory markers were rarely expressed in CD8^+^ T cell clusters and most of them were naive cells (Supplemental Figure 2E). PCA and sample-to-sample distance analysis of CD8^+^ T cells in kidneys and spleens from accepted, rejecting, and naive mice showed that accepted kidney samples at 1 and 3 weeks clustered with accepted recipient spleen at 24 weeks and not with rejecting kidney allograft samples (Supplemental Figure 2, F and G).

ELISPOT assay analysis showed that the production of IFN-γ by isolated CD8^+^ T cells from renal grafts at 24 weeks after transplantation was significantly lower in response to donor antigen when compared with CD8^+^ T cells isolated from recipient spleen (Figure 2F) and to total T cells (Figure 2F).

To further investigate whether these gene expression patterns are unique to acceptance, we compared CD8^+^ T cell clusters in scRNA-seq data from accepted and rejected kidney allografts at 1 week after transplantation, showing that intragraft CD8^+^ T cells express more cytotoxic markers (*Gzmb*, *Ifng*, and *Prf1*) than CD8^+^ T cells isolated from accepted kidneys (Figure 2G). These results support our previous report (5) in bulk RNA analysis, which showed that *Prf1* expression was significantly higher in rejected kidney allografts than in accepted ones at 1 week. In addition, there was greater expression of *Pdcd1* in CD8^+^ T cells from rejecting kidneys, as well as expression of other exhaustion and regulatory markers (*Tox*, *Lag3*, *Fgl2*, and *Il2rb*). However, 3 weeks after transplantation, we observed greater gene expression in the acceptance samples of exhausted and regulatory markers (*Fgl2* and *Il2rb*), including *Pdcd1* (Figure 2G).

Heatmap analysis of CD8^+^ T cell clusters showed the top differentially expressed genes at each time point after transplantation (Figure 2H). At 1 week, gene markers of proliferation (*Mki67*, *Top2a*, and *Stmn1*), cell cycle progression (*Birc5* and *Pclaf*), and chromatin binding (*Hist1h1b*, *Hist1h1e*, and *Plac8*) were some of the highest expressors. However, at 3 weeks, gene markers of CD8^+^ Tregs (*Fgl2* and *Il2rb*), tissue-resident memory T cells (*Itga1*, *Itgae*, *Cxcr6*, and *Runx3*), effector memory T cells (*Ccl5*, *Cd7*, and *Id2*), and genes associated with the apoptosis regulator (*Bcl2*) represented the highest expression. By 24 weeks, gene markers of naive cells (*Lef1*, *Klf2*, *Satb1*, *Sell*, *Tcf7*, and *Ccr7*) and central memory T cells (*Sell* and *Il7r*) dominated the pool of highest expressors. Volcano plot analysis of CD8^+^ T cell clusters between 1-week, 3-week, and 24-week time points showed that gene expression of regulatory genes (*Il2rb*, *Fgl2*, *Bcl2*, and *Btg1*) and markers of tissue-resident memory T cells (*Itgae*, *Itga1*, and *Runx3*) were increased by 3 weeks when compared with 1 week (Supplemental Figure 3A). At 1 week, genes related to proliferation (*Mki67*, *Top2a*, *Pclaf*, and *Stmn1*) and cytotoxicity (*Nme2*) were elevated, as well as regulatory genes (*Ppia*, *Ran*, and *Tpt1*). Comparison of 3 and 24 weeks showed that regulatory/exhaustion–related genes (*Pdcd1*, *Fgl2*, and *Il2rb*) were more elevated at 3 weeks, while marker genes of naive phenotype and central memory cells (*Lef1*, *Klf2*, *Sell*, and *Tcf7*) were upregulated at 24 weeks (Supplemental Figure 3B). The renal CD8^+^ T cell population signatures at 1 and 3 weeks show similarity to the CD8^+^ T cell signature in 24-week recipient spleen; 24-week CD8^+^ T cells from accepted kidney samples showed similarity to naive, 1-, and 3-week recipient spleen samples (Supplemental Table 1 and Supplemental Figure 4, A–H).

### CD8^+^CD122^+^ T cells from accepted kidney allografts exhibit suppressive function.

We investigated the regulatory function of CD8^+^CD122^+^ T cells isolated from accepted kidney allografts for their ability to suppress proliferation of CD4^+^ T cells in vitro. FACS-isolated CD8^+^CD122^+^ T cells, CD8^+^CD122^–^ T cells, and CD4^+^CD25^+^ Tregs, cocultured with CellTrace Violet–labeled CD4^+^CD25^–^ T cells at various ratios, revealed that cells sorted from accepted kidney allografts significantly suppressed naive CD4^+^ T cell proliferation in a dose-dependent manner (suppressor/responder ratio = 2:1, 27.5%; 1:1, 23.5%; 0:1, 17.1%), although less effectively than CD4^+^CD25^+^ Tregs isolated from naive spleen (suppressor/responder ratio = 2:1, 60.8%; 1:1, 45.3%) (Supplemental Figure 5, A and B).

### CD8^+^ T cells are reprogrammed to an exhausted/regulatory-like phenotype in the graft.

**Figure 3:**
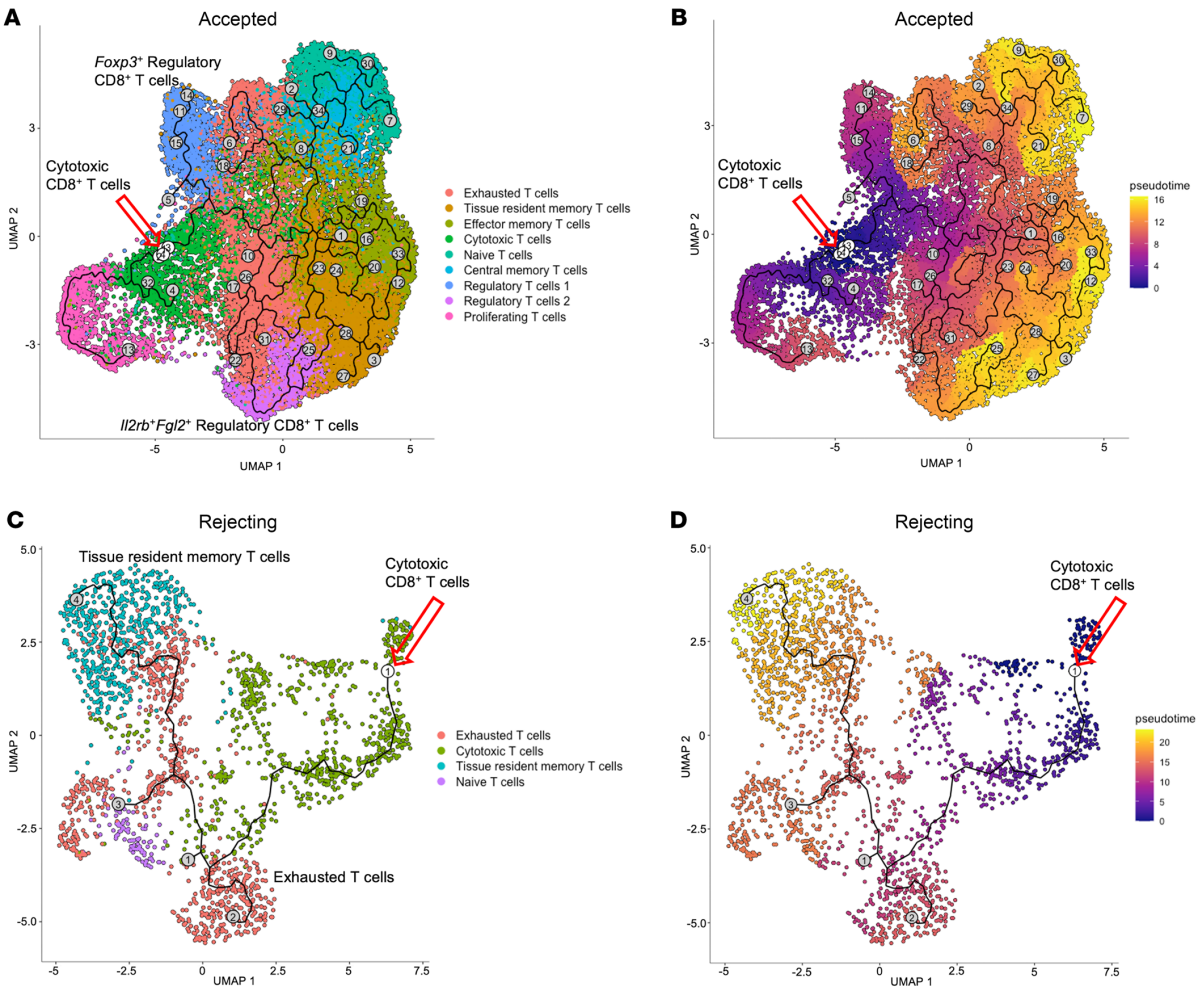
CD8^+^ T cells are reprogrammed to an exhausted/regulatory-like phenotype in the graft. (**A**–**D**) Trajectory analysis of CD8^+^ T cells in accepted kidney allografts (**A** and **B**) and rejecting kidney allografts (**C** and **D**). (**A** and **C**) Monocle 3 map illustrating trajectory nodes and origin cells (cytotoxic CD8^+^ T cells) on the CD8^+^ T cell data set. The red arrow points to nodes within the Monocle data set chosen as the origin cells, represented by the cytotoxic CD8^+^ T cell population. Gray circles represent termination states of cell trajectories. (**B** and **D**) Pseudotime analysis for CD8^+^ T cell populations. The red arrow pointing to the white circle represents the origin point (cytotoxic CD8^+^ T cells). Gray circles represent termination states of cell trajectories. Pseudotime is overlaid on the UMAP plot on a gradient color scale (purple to yellow representing less time to longer time needed to reach a given state, respectively).

To investigate the possibility of intragraft reprogramming of cytotoxic CD8^+^ T cells to an exhausted/regulatory state, we performed trajectory analysis of the scRNA-seq data using Monocle 3 (17–19), with a focus on the CD8^+^ T cell subcluster in accepted kidney allografts. This software platform allows for determining terminal cellular states, intermediate states, and potential starting states within the transplanted allografts. To perform an accurate trajectory analysis of T cells within the transplanted allograft, “cells of origin” were selected in Monocle 3 (17–19) (Figure 3A, indicated by red arrow). Using the cytotoxic CD8^+^ T cell cluster as the point of origin, trajectory analysis of the integrated scRNA-seq data set revealed multiple branches (black line) and termination points (gray circles). Cytotoxic CD8^+^ T cells evolved into proliferating T cells, exhausted T cells, and finally into *Il2rb*^+^*Fgl2*^+^ Tregs, tissue-resident memory T cells, effector memory T cells, and central memory T cells within the accepted kidney allografts (Figure 3A). Pseudotime analysis represents how quickly or slowly the cell type transitions to other states. Overlaying the pseudotime graphic with the UMAP of T cell subtypes, the transition from cytotoxic CD8^+^ T cells to exhausted CD8^+^ T cells occurred before the transition to *Fgl2*^+^*Il2rb*^+^ CD8^+^ Tregs, tissue-resident memory T cells, and effector memory T cells (Figure 3B). We next performed trajectory analysis of scRNA-seq data from rejecting kidney allografts and used cytotoxic CD8^+^ T cells as a point of origin. Although we found that cytotoxic CD8^+^ T cells evolved into exhausted T cells and tissue-resident memory T cells within a week, they did not evolve into Tregs (Figure 3, C and D).

### Increased IFN-γ expression and production within the T cell population in accepted kidney allografts.

**Figure 4:**
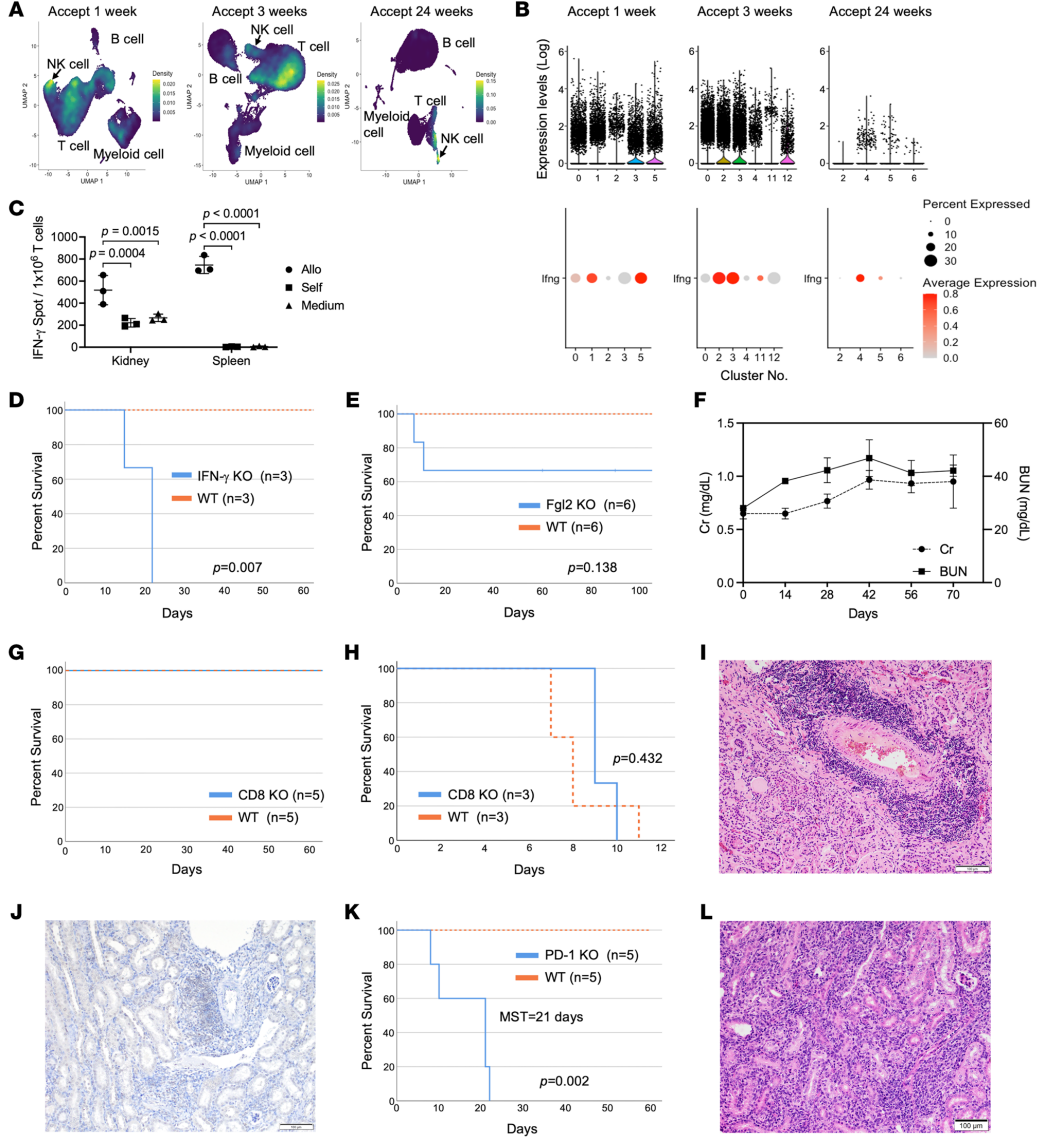
Increased IFN-γ expression and production within the T cell population in accepted kidney allografts, but FGL2 and CD8^+^ cells are not needed for the induction and maintenance of accepted kidney allografts. (**A**) Density plots of *Ifng*^+^ cells in total cells in accepted kidney allografts at each time point. (**B**) Violin plots and dot plots show levels and percentages of *Ifng* expression in T cell subset in accepted kidney allografts at each time point. (**C**) Bar graph shows ELISPOT analysis of IFN-γ production in T cells obtained from accepted kidney allograft and recipient’s spleen at 4 weeks after transplantation. Data are represented as mean ± SD, compared by 2-way ANOVA test. (**D**) Graft survival curve after kidney transplantation into IFN-γ–KO (*n* = 3) or WT (*n* = 3) mice (*P* = 0.007). (**E**) Graft survival curve after kidney transplantation into Fgl2-KO (*n* = 6) or WT (*n* = 6) mice (*P* = 0.138). (**F**) Line graph of serum levels of creatinine (Cr) and blood urea nitrogen (BUN) in long-term-surviving Fgl2-KO recipients (*n* = 3). Data represent the mean ± SEM. (**G**) Graft survival curve after kidney transplantation into CD8-KO (*n* = 5) and WT (*n* = 5) mice. (**H**) Graft survival curve after heart transplantation into CD8-KO (*n* = 3) and WT (*n* = 3) mice (*P* = 0.432). (**I** and **J**) Pathological findings of kidney allografts obtained from CD8-KO recipients. (**I**) H&E staining shows perivascular rTLO formation. (**J**) Immunohistochemistry of CD8 staining shows the absence of CD8^+^ cell infiltration in kidney allografts taken from CD8-KO recipients. (**K**) Graft survival curve after kidney transplantation into PD-1–KO (*n* = 5) and WT (*n* = 5) mice (*P* = 0.002). (**L**) H&E staining of kidney allografts obtained from PD-1–KO mice shows signs of rejection. Statistical significance was determined by log-rank test (**D**, **E**, **H**, and **K**). Scale bars: 100 μm (**I**, **J**, and **L**).

Our scRNA-seq data showed that *Fgl2* is markedly elevated in graft-infiltrating cytotoxic CD8^+^ T cells by 3 weeks after transplantation, reflective of a regulatory-like phenotype. It has been shown that *Fgl2* expression can be driven by exposure to several cytokines, such as TNF-α and IFN-γ (20–22). Analysis of scRNA-seq data from the different time points after transplantation showed an increase in *Ifng* expression within the T cell and NK cell clusters (Figure 4, A and B). ELISPOT analysis of T cells isolated from accepted kidneys showed increased production of IFN-γ even in media-alone samples, which suggested that IFN-γ is actively produced by cells in tolerated kidney allografts even without in vitro antigen stimulation (Figure 4C). T cells from recipient spleens did not secrete IFN-γ in media alone (Figure 4C).

The importance of IFN-γ production in our kidney acceptance model was further assessed in DBA/2J kidneys transplanted into B6.*Ifng*^–/–^ (IFN-γ–KO) recipients (*n* = 3). These kidney allografts were rejected with an MST of 22 days (*P* = 0.007) (Figure 4D). Similar observations have been reported by Mele and colleagues when looking at spontaneous acceptance of liver allografts in IFN-γ–KO recipients (23). Our data suggest that IFN-γ is required to maintain kidney allograft acceptance. We hypothesize that sustained production of IFN-γ creates an intragraft environment favorable for the increased expression of *Fgl2* within infiltrating CD8^+^ T cells.

### FGL2 and CD8^+^ cells are not needed for the induction and maintenance of accepted kidney allografts.

Fgl2-KO mouse recipients were used to test the functional significance of the increased expression of *Fgl2*. Figure 4E shows that 2 out of 6 Fgl2-KO recipients rejected their kidney allografts, while 4 out of 6 survived over 60 days (Figure 4E) without rejection or renal dysfunction (Figure 4F). These data suggest that FGL2 is not essential for induction and maintenance of kidney allograft tolerance. We have shown that CD8^+^ T cells infiltrate the grafts as cytotoxic T cells, but are reprogrammed in situ to an exhausted/regulatory-like phenotype. This reprogramming would suggest that CD8^+^ T cells may not play a role in the induction and maintenance of kidney allograft tolerance. To test this, we transplanted DBA/2J kidneys into CD8-KO mouse recipients. CD8-KO mice reject skin and heart allografts (24). DBA/2J kidneys transplanted into CD8-KO B6 recipients were accepted long term (over 90 days) (Figure 4G), while DBA/2J heart allografts were rejected at similar rates in CD8-KO and WT recipients (Figure 4H). Kidney allografts obtained from CD8-KO recipients showed normal rTLO formation, with no signs of rejection (Figure 4I) and the absence of CD8^+^ cells (Figure 4J). These data show that CD8^+^ cells are not needed for kidney allotransplant tolerance.

### PD-1 is required for the maintenance of accepted kidney allografts.

To investigate whether T cell exhaustion plays a key role in kidney allotransplant tolerance, we transplanted renal grafts into B6.PD-1–KO recipients. In Figure 4K, we show that B6.PD-1–KO recipients rejected DBA/2J kidney allografts, with an MST of 21 days (*P* = 0.002). Pathological findings confirmed acute cellular rejection without rTLO formation (Figure 4L). These results suggest that the induction and maintenance of tolerance is lost in the absence of PD-1.

### Reprogramming of donor-reactive T cells following adoptive transfer in the presence of an accepted kidney allograft.

**Figure 5:**
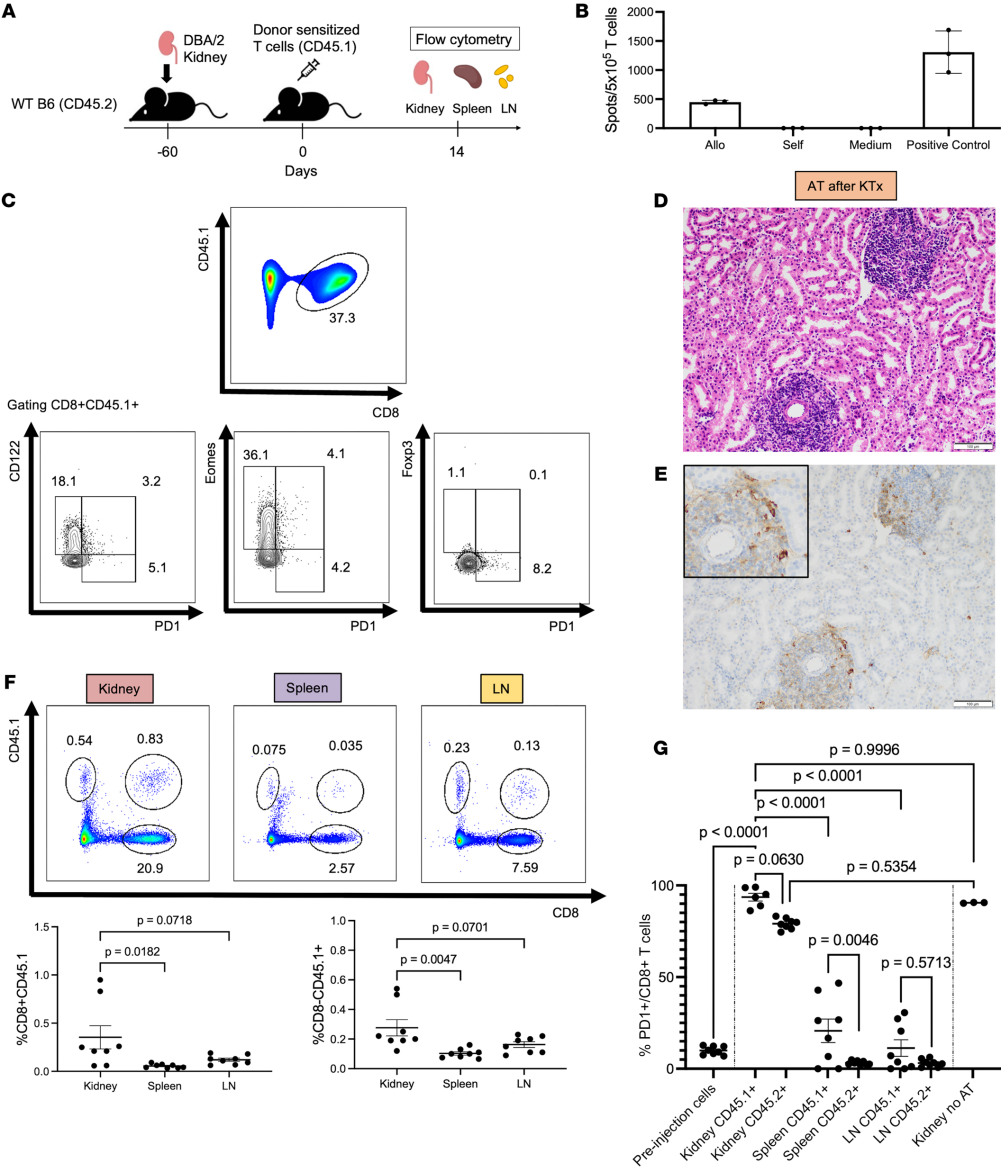
Reprogramming of donor-reactive T cells following adoptive transfer in the presence of an accepted kidney allograft. (**A**) Schematic of experimental design. (**B**) ELISPOT assay of donor-sensitized T cells prior to transfer (*n* = 3). Data represent the mean ± SD. (**C**) Frequency of CD8^+^ T cells in donor-sensitized T cells and levels of PD-1, CD122, Eomes, and Foxp3 expression in CD8^+^CD45.1^+^ donor-sensitized T cells prior to transfer. (**D** and **E**) Pathological findings of kidney allografts collected from recipient mice that underwent adoptive transfer (AT) after kidney transplantation (KTx) at 14 days after AT. H&E staining (**D**) and CD45.1 immunohistochemical staining (**E**). Scale bars: 100 μm. (**F**) Frequency of CD8^+^CD45.1^+^ T cells and CD8^–^CD45.1^+^ T cells in kidney, spleen, and lymph node (LN) obtained from recipients that underwent AT after KTx. (**G**) Frequency of the percentage of PD-1^+^ cells per CD8^+^CD45.1^+^ adoptively transferred cells and CD8^+^CD45.2^+^ recipient endogenous cells. Data in **F** and **G** are represented as mean ± SEM, compared by 1-way ANOVA with Tukey’s post hoc test for multiple comparisons.

The next question is whether accepted kidney allografts can reprogram sensitized effector cytotoxic CD8^+^ T cells to provide a defense against tissue damage by converting pro-rejection immune cells to a benign, pro-tolerance cell type. Russell and colleagues previously reported that adoptive transfer of donor-sensitized cells did not cause rejection of tolerated kidneys, although they caused a transient renal dysfunction (15). Here, donor-sensitized T cells were isolated from the spleen of CD45.1 B6 recipients of DBA/2J skin grafts. The alloreactive T cells were adoptively transferred into kidney transplant CD45.2 B6 recipients that received DBA/2J kidney allografts 2 months earlier (Figure 5A). Donor alloreactivity of CD45.1^+^ T cells was confirmed by ELISPOT before adoptive transfer (Figure 5B). Flow cytometric analysis showed that the CD8^+^ T cell population represented 37% of total T cells, and 10% of CD8^+^ T cells were PD-1^+^ prior to adoptive transfer (Figure 5C).

In this experiment, the serum BUN and creatinine levels did not increase, but were maintained within normal range 14 days after adoptive transfer of sensitized cells (Supplemental Figure 6A). Histology showed fully formed rTLOs and no histologic signs of rejection (Figure 5D), similar to accepted kidney allografts that received no alloreactive T cells (4, 5). Remarkably, immunohistochemistry showed CD45.1^+^ cells mostly localized within the rTLOs of CD45.2 kidney allografts (Figure 5E).

T cells were purified from kidney allografts, spleens, and lymph nodes at a median time of 14 days after adoptive cell transfer. Flow cytometry showed the transferred cells migrated selectively into kidney allografts (0.35% of viable lymphocytes), compared with the spleen and lymph nodes (0.05% [*P* = 0.0182] and 0.12% [*P* = 0.0718], respectively) (Figure 5F). The percentage of CD8^+^CD45.1^+^ cells expressing PD-1 was significantly higher in the kidney allografts (93.6%) compared with spleen (20.7%, *P* < 0.0001), lymph node (11.3%, *P* < 0.0001), or the alloreactive cells prior to being adoptively transferred (9.1%, *P* < 0.0001) (Figure 5G). CD8^+^CD45^+^ cells isolated from DBA/2J kidney allografts at 1 week after transplantation that did not receive alloreactive donor cells had a similar level of PD-1 expression (90.5%, *P* = 0.9996) (Figure 5G). The increase in PD-1 expression in alloreactive CD8^+^ T cells infiltrating the kidney allograft occurred within 2 weeks, similar to the peak expression of *Pdcd1* at 3 weeks after transplantation in scRNA-seq analysis of the accepted kidney allografts.

Recipient-endogenous CD8^+^CD45.2^+^ T cells isolated from the kidney allografts represented 15.8% of viable lymphocytes, of which 79.1% were PD-1^+^ and less than CD8^+^CD45.1^+^ transferred T cells (*P* = 0.0630). In contrast, recipient-endogenous CD8^+^ T cells in the spleen and lymph nodes represented 4.2% and 10.7% of the viable lymphocytes, and only 3.2% and 3.1% of them were PD-1^+^, respectively (Figure 5, F and G).

### Reprogramming of donor-reactive T cells does not occur in the absence of an accepted kidney allograft.

The observation that adoptively transferred alloreactive T cells did not cause rejection in the presence of an already accepted kidney allograft suggests that cytotoxic T cells are being reprogrammed to an exhausted/regulatory-like phenotype that does not mediate tolerance, but permits it by inactivity. Here, we asked whether the presence of a kidney allograft was necessary for cytotoxic-to-exhausted/regulatory-like CD8^+^ T cell reprogramming.

**Figure 6:**
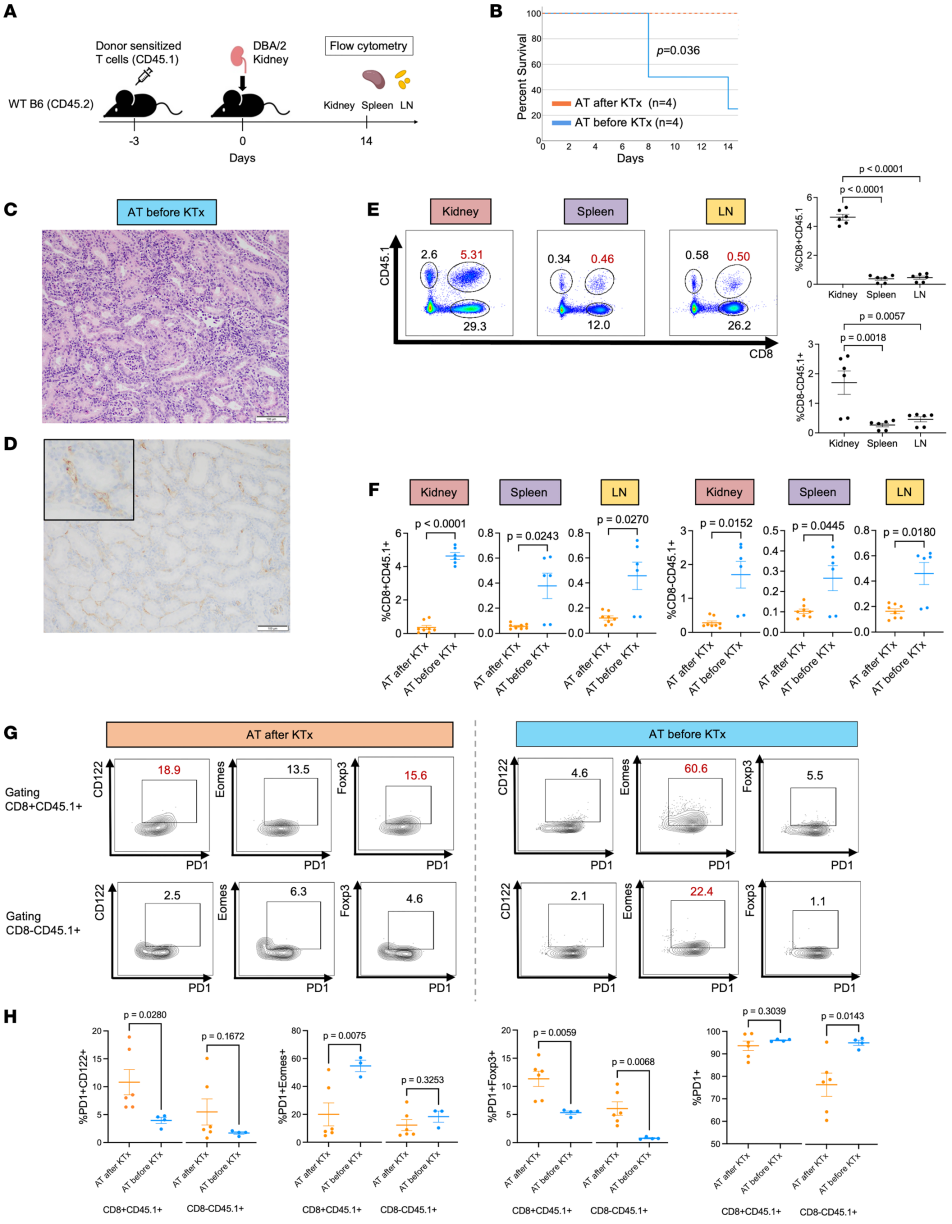
Reprogramming of donor-reactive T cells does not occur in the absence of an accepted kidney allograft. (**A**) Schematic of experimental design. (**B**) Graft survival curve of kidney transplantation in the setting of adoptive transfer (AT) before kidney transplantation (KTx) (*n* = 4) or AT after KTx (*n* = 4). Log-rank test, *P* = 0.036. (**C** and **D**) Pathological findings of kidney allografts obtained from recipients that underwent adoptive transfer (AT) before kidney transplantation (KTx). H&E staining (**C**) and CD45.1 immunohistochemical staining (**D**). Scale bars: 100 μm. (**E**) Frequencies of CD8^+^CD45.1^+^ T cells and CD8^–^CD45.1^+^ T cells in kidney, spleen, and lymph node (LN) obtained from recipients that underwent AT before KTx. Data are represented as mean ± SEM, compared by 1-way ANOVA with Tukey’s post hoc test for multiple comparisons. (**F**) Frequency of the percentage of CD8^+^CD45.1^+^ cells and CD8^–^CD45.1^+^ cells per viable lymphocyte in kidney, spleen, and LN was compared between AT after KTx and AT before KTx. (**G**) Frequencies of PD-1^+^CD122^+^, PD-1^+^Eomes^+^, and PD-1^+^Foxp3^+^ cells per CD8^+^CD45.1^+^ T cell and CD8^–^CD45.1^+^ T cell in accepted kidney allografts obtained from AT after KTx and rejecting kidney allografts from AT before KTx were analyzed. (**H**) Frequencies of PD-1^+^CD122^+^, PD-1^+^Eomes^+^, PD-1^+^Eoxp3^+^, and PD-1^+^ cells per CD8^+^CD45.1^+^ and CD8^–^CD45.1^+^ cell were compared between accepted kidney allografts obtained from AT after KTx and rejecting kidney allografts from AT before KTx. Data in **F** and **H** are represented as mean ± SEM, compared by 2-tailed Student’s *t* test.

To test this, we adoptively transferred donor-sensitized CD45.1^+^ cells into CD45.2 WT.B6 recipients 3 days prior to the transplantation of DBA/2J kidneys (Figure 6A). We showed that the transfer of alloreactive cells before a kidney is transplanted and developed rTLOs resulted in the rejection of 3 out of 4 allografts, with an MST of 8 days after transplantation (*P* = 0.036) (Figure 6B) and elevated BUN levels over 100 mg/dL (Supplemental Figure 6B). Histological analysis confirmed cellular graft rejection without rTLO formation (Figure 6C). Immunohistochemical staining with anti-CD45.1 antibodies showed a diffuse infiltrate of CD45.1^+^ cells within the graft (Figure 6D). This diffuse distribution of CD45.1-alloreactive cells contrasts with the localized distribution of CD45.1^+^ cells specifically in rTLOs when alloreactive cells were transferred into recipients 8 weeks after kidney transplantation (Figure 5E).

Recipients of adoptively transferred cells before kidney transplantation showed a significantly higher number of CD8^+^CD45.1^+^ and CD8^-^CD45.1^+^ T cells migrating in kidney allografts, spleen, and lymph nodes (CD8^+^CD45.1^+^: 4.64%, 0.38%, and 0.46%; CD8^-^CD45.1^+^: 1.70%, 0.27%, and 0.46%, respectively) than in recipients with a preexisting kidney transplant (Figure 6, E and F). Further analysis showed that CD8^+^CD45.1^+^ T cells infiltrating the kidney allografts following adoptive transfer before and after transplantation differed in CD122, Eomes, and Foxp3 expression. CD8^+^CD45.1^+^ cells infiltrating the kidney allograft were shown to express more CD122 and Foxp3 if the alloreactive T cells were adoptively transferred after transplantation versus if transferred before transplantation (10.8% and 11.3% versus 3.9% and 5.3%, *P* = 0.0280 and *P* = 0.0059, respectively) (Figure 6, G and H). Interestingly, if alloreactive cells were transferred before transplantation, infiltrating CD8^+^CD45.1^+^ cells showed greater Eomes expression when compared with alloreactive cells transferred after transplantation (54.7% versus 20.0%, *P* = 0.0075) (Figure 6, G and H). Expression of PD-1 was comparable between the 2 groups (93.6% versus 96.8%, *P* = 0.3039) (Figure 6H).

### Reprogramming of donor-reactive T cells does not occur in alloreactive T cells that lack IFN-γ receptor.

In Figures 5 and 6, we showed that adoptively transferring alloreactive T cells into a kidney allograft recipient did not cause rejection, but if alloreactive cells were adoptively transferred before the kidney was transplanted, rejection of the subsequent renal transplant occurred, suggesting that ongoing tolerance is required if the reprogramming of cytotoxic T cells to exhausted/regulatory-like cells is to occur. In addition, the observation that IFN-γ production was increased in renal allografts, as assessed by both scRNA-seq and ELISPOT (Figure 4, B and C), and that kidney transplantation into B6.IFN-γ–KO recipients resulted in rejection (Figure 4D), suggested to us a possible role of IFN-γ in the reprogramming of cytotoxic to exhaustion/regulatory-like CD8^+^ T cells.

**Figure 7:**
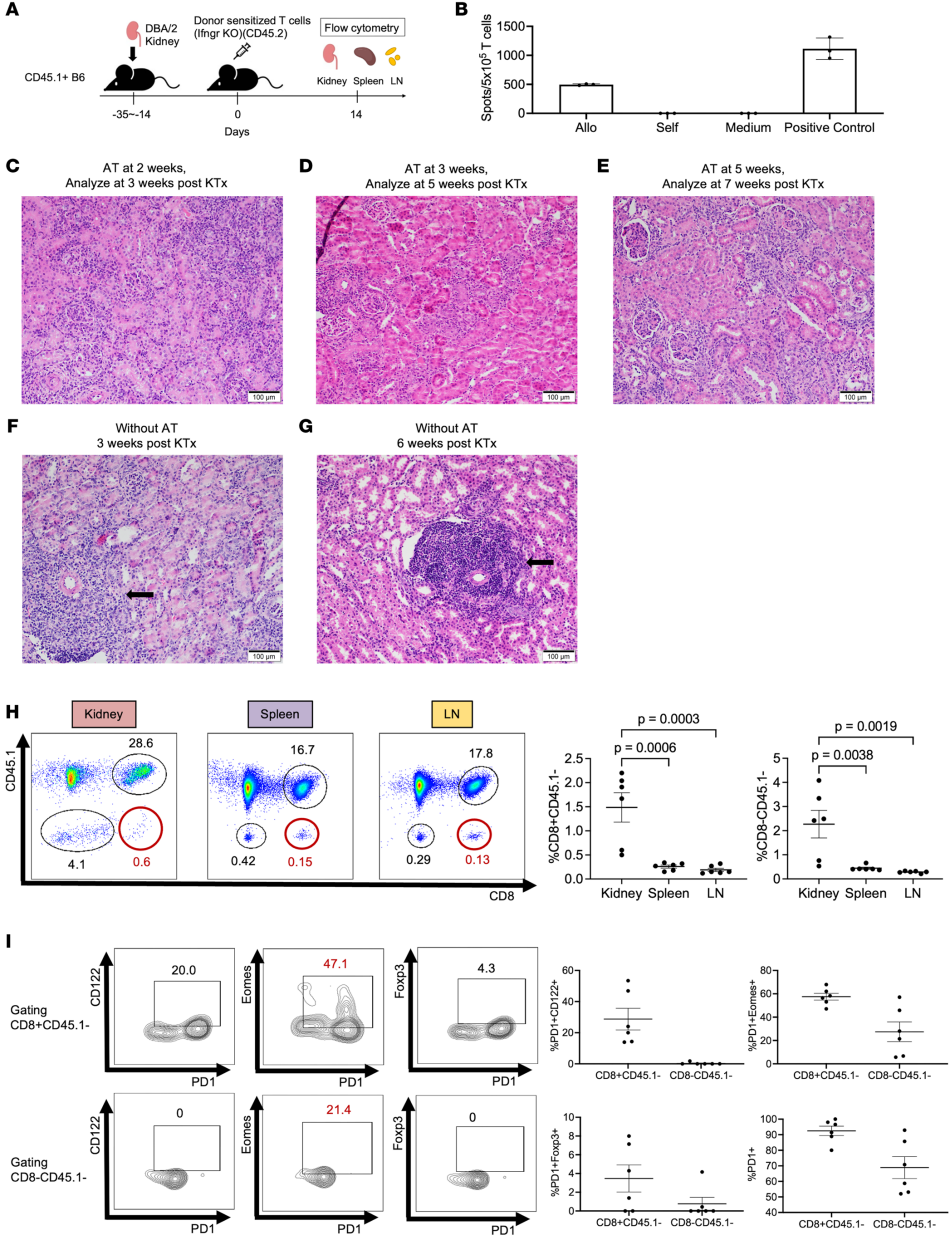
Reprogramming of donor-reactive T cells does not occur in alloreactive T cells that lack the IFN-γ receptor (IFNGR). (**A**) Schematic of experimental design. (**B**) ELISPOT assay of donor-sensitized T cells prior to transfer (*n* = 3). Data represent the mean ± SD. (**C**–**G**) Pathological findings of kidney allografts collected from recipients that underwent adoptive transfer (AT) after kidney transplantation (KTx) at 2 to 5 weeks after transplantation (**C**–**E**) and from WT.B6 without AT at 3 weeks (**F**) and 6 weeks after KTx (**G**). H&E staining (**C**–**G**). The black arrow indicates rTLOs (**F** and **G**). Scale bars: 100 μm. (**H**) Frequency of CD8^+^CD45.1^–^ T cells and CD8^–^CD45.1^–^ T cells in kidney, spleen, and lymph node (LN) obtained from recipients that underwent AT of IFNGR-KO alloreactive cells after KTx. Data are represented as mean ± SEM, compared by 1-way ANOVA with Tukey’s post hoc test for multiple comparisons. (**I**) Frequency of PD-1^+^CD122^+^, PD-1^+^Eomes^+^, and PD-1^+^Foxp3^+^ cells per CD8^+^CD45.1^–^ T cell and CD8^–^CD45.1^–^ T cell in rejecting kidney allografts from recipients that underwent AT of IFNGR-KO alloreactive cells after KTx. Data are represented as mean ± SEM.

To test this, we adoptively transferred donor-sensitized T cells that lack the IFN-γ receptor (CD45.2^+^ IFNGR-KO T cells) into CD45.1 B6 kidney allograft recipients that received DBA/2J kidney allografts 2 to 5 weeks earlier (Figure 7A). The donor alloreactivity of IFNGR-KO T cells was assessed by ELISPOT prior to adoptive transfer (Figure 7B).

To be consistent with the experiment outlined in Figure 5D, in which WT alloreactive T cells were adoptively transferred and the kidney graft was isolated at 14 days to assess pathology, a similar experiment was performed following the adaptive transfer of alloreactive IFNGR-KO T cells. Although the serum BUN and creatinine levels did not increase and were maintained within normal range 14 days after adoptive transfer of alloreactive T cells, histologic images of kidney allografts after adoptive transfer of donor-sensitized T cells showed signs of acute cellular rejection beginning at 2 weeks, characterized by multiple foci of interstitial inflammation, marked tubulitis, and the disruption of rTLOs (Figure 7, C–E). Glomerulitis was also present and was more prominent in adoptive transfer at 5 weeks after transplantation, suggesting an antibody-mediated component (H&E, ×200) (Figure 7E). Untreated kidney allografts at the same time point after transplantation showed intact rTLOs and no signs of rejection (Figure 7, F and G), similar to what was observed with adoptive transfer of WT alloreactive T cells (Figure 5D).

Flow cytometry showed the transferred CD8^+^ T cells migrated selectively into the kidney allografts (1.48% of viable lymphocytes), compared with the spleen and lymph nodes (0.26% and 0.19%, respectively) (Figure 7H). In addition, infiltrating CD8^+^ T cells from kidney allografts show higher levels of Eomes expression (Figure 7I), consistent with what we observed in rejecting grafts when alloreactive cells were transferred before transplantation (Figure 6, G and H).

### The T cell population in pancreatic and colorectal tumors also exhibits Cd8^+^Fgl2^+^Il2rb^+^ cells, similar to tolerant allografts.

**Figure 8:**
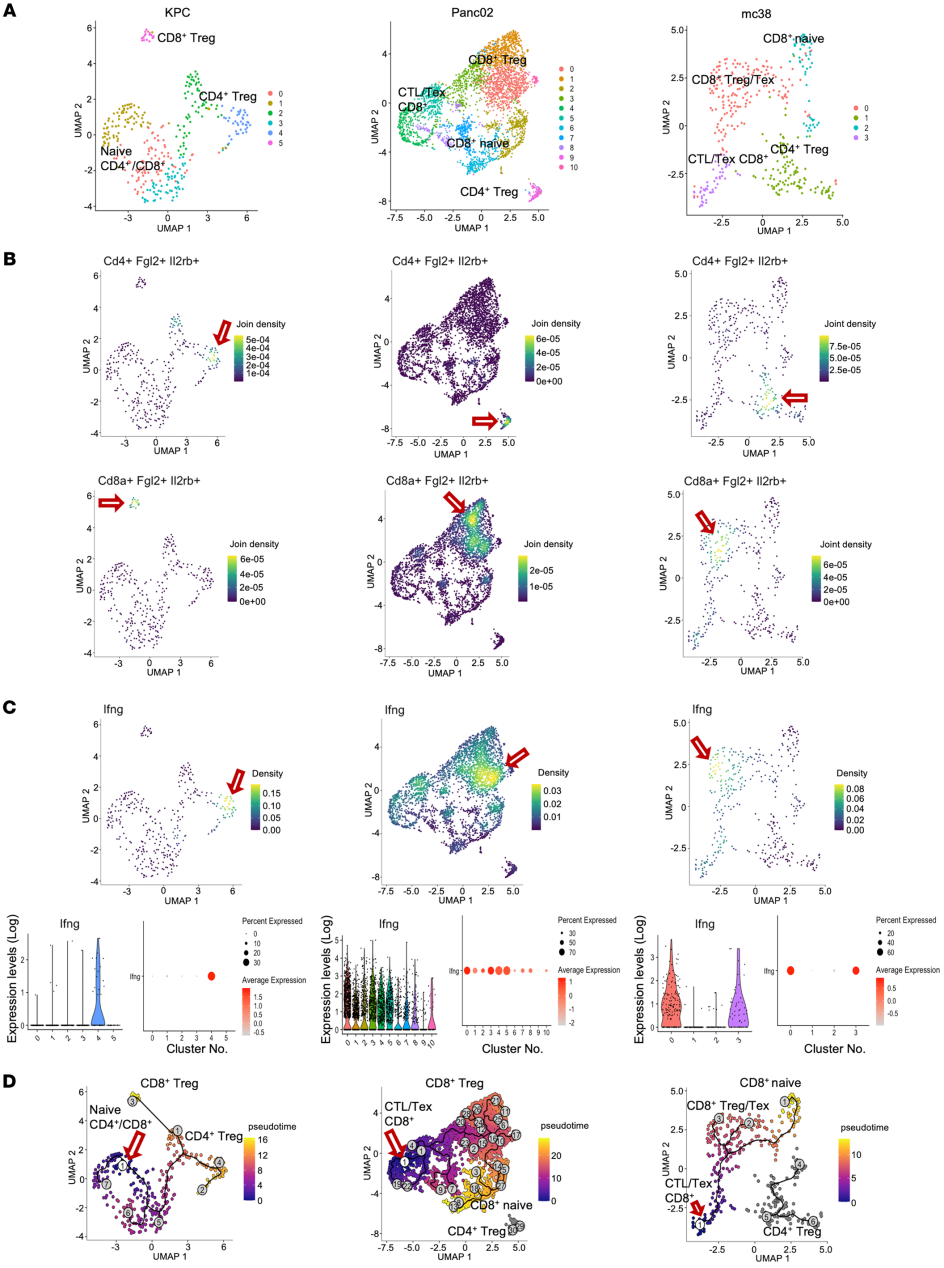
The T cell population in pancreatic and colorectal tumors also exhibits *Cd8*^+^*Fgl2*^+^*Il2rb*^+^ cells, similar to tolerant allografts. (**A**) UMAP plots of T cell clusters in KPC tumor– (left), Panc02 tumor– (middle), and mc38 tumor–infiltrated (right) immune cells (see also Supplemental Figure 7, A and B). (**B**) Density plots of *CD4^+^Fgl2^+^Il2rb^+^* (top) and *CD8^+^Fgl2^+^Il2rb^+^* (bottom) expression. Red arrows indicate high-density areas. (**C**) Density plot, violin plot, and dot plot show the *Ifng* expression in T cell clusters. (**D**) Pseudotime analysis of T cell clusters. The red arrow marks the point of origin (white circle) — naive cells for KPC tumors, and cytotoxic CD8^+^ T cells for Panc02 and mc38 tumors. Pseudotime graphic is overlaid on the UMAP plot on a gradient color scale. Pancreatic cancer cell (Panc02-SIY) data sets GSM6048775, GSM6048776, GSM6048777, and GSM6048778 contained in data set GSE201026, and colon cancer cell (mc38) data sets GSM5460383, GSM5460384, GSM5460385, and GSM5460386 contained in data set GSE180296, were downloaded from the NCBI Gene Expression Omnibus.

While accepted kidneys develop rTLOs that may function to maintain tolerance to the graft, resulting in long-term survival, the tumor immune microenvironment is defined by the development of TLSs (25–27), similar to rTLOs found in kidney allografts and may serve a similar function — in other words, maintaining tolerance to the tumor. Therefore, we sought to define whether *Cd8*^+^*Fgl2*^+^*Il2rb^+^* cells, similar to those in accepted kidney allografts, could be found within the immune cell population in tumors. In Figure 8, we summarize the scRNA-seq data derived from 3 independent, spontaneous mouse tumors (2 pancreatic and 1 colorectal) (Supplemental Figure 7A). We observed that not only were the T cell clusters made up of exhausted T cells and Treg, but we also observed the presence of *Cd8*^+^*Fgl2*^+^*Il2rb^+^* cells (Figure 8, A and B and Supplemental Figure 7B) and increased *Ifng* expression (Figure 8C and Supplemental Figure 7B), similar to cells in tolerated kidney allografts. Interestingly, *Ifng*-expressing cells were densely located in Treg clusters, and their distribution was similar to that of *Fgl2^+^Il2rb^+^* cells. We performed trajectory analysis of the scRNA-seq data via Monocle 3 (17–19) to determine whether intratumor cytotoxic CD8^+^ T cells were also reprogrammed to an exhausted/regulatory state. Since no cytotoxic CD8^+^ T cells were identified in the scRNA-seq data set for KPC tumor, we used naive CD4^+^ and CD8^+^ T cells as the point of origin for our analysis for that sample and found that these cells evolved into CD4^+^ and CD8^+^ Tregs within the tumor (Figure 8D). Using cytotoxic CD8^+^ T cells as the point of origin for Panc02 and mc38 tumors, these cells evolved into CD8^+^ exhausted T cells, Tregs, and finally, naive cells (Figure 8D). PCA and sample-to-sample distance analyses comparing tumor data sets with kidney and spleen data sets revealed that 2 of the 3 tumor data sets were strongly associated with accepted kidney allografts at 1 to 3 weeks after transplantation. One tumor data set, in which naive and central memory cells were the major cell populations, was associated with accepted kidney allografts at 24 weeks after transplantation (Supplemental Figure 7, C and D).

## Discussion

Our previous studies have shown that, in certain murine strain combinations, kidney allografts are accepted without the use of immunosuppressive drugs (4, 5, 7, 15, 28). These accepted allografts are characterized by the presence of TOLS, novel regulatory TLOs that form perivascularly and comprise various immune cells (4–6). We have also shown through bulk mRNA and scRNA-seq analyses that CD8^+^ T cells were the abundant cell type early after transplantation (i.e., at 1 and 3 weeks) and then decreased with time (i.e., by 6 months), while the B cell signature increased and Tregs remained constant (5, 6). These findings were corroborated by flow cytometric and immunohistological analyses (5, 6).

In the current study, we more specifically characterized the infiltrating CD8^+^ T cell population using scRNA-seq on CD45^+^ sorted cells isolated from accepted or rejecting renal allografts at 1 week, and accepted kidneys at 3 weeks and 24 weeks after transplantation. scRNA-seq data revealed, in accepted kidneys, a temporal shift of kidney allograft–infiltrating CD8^+^ T cells from a cytotoxic phenotype at 1 week to an exhausted/regulatory-like one by 3 weeks after transplantation. We have termed this process “defensive tolerance.”

The current study revealed that cytotoxic markers (*Gzmb* and *Ifng*) and proliferating T cell markers (*Mki67* and *Top2a*) were highly expressed within the CD8^+^ T cell population as early as 1 week after transplantation. These transcripts then decreased by 3 weeks. At the same time, the expression of exhaustion/regulatory markers (*Fgl2*, *Il2rb*, *Pdcd1*, *Tox*, and *Lag3*) and effector memory CD8^+^ T cell markers (*Id2* and *Ccl5*) were significantly increased. In addition, tissue-resident memory T cell markers (*Itga1*, *Itgae*, *Cxcr6*, and *Runx3*) were significantly elevated at 3 weeks. By 24 weeks, expression of cytotoxic and exhaustion/regulatory markers were reduced. However, memory and naive T cell markers (*Lef1*, *Klf2*, *Sell*, *Ccr7*, *Il7r*, and *Tcf7*) were highly expressed. Trajectory analysis confirmed the intragraft reprogramming of infiltrating cytotoxic CD8^+^ T cells to exhausted/regulatory-like CD8^+^ T cells. In contrast, the CD8^+^ T cell cluster in the recipient spleen expressed regulatory and exhausted markers only at 24 weeks. Neither cytotoxic nor exhaustion/regulatory markers were expressed in the naive spleen.

One of the genes found to be highly expressed at 3 weeks after transplantation, especially within the CD8^+^ T cell cluster, is *Fgl2*. This gene encodes fibrinogen-like protein 2 and has been shown to be secreted by CD8^+^ Tregs (10, 11). In the present study, *Fgl2* expression was notably elevated in accepted kidney allografts at 3 weeks after transplantation when rTLOs were fully formed, while in the spleen, it was barely elevated in the early stages and only late in the process. However, 4 out of 6 Fgl2-KO recipients did not reject their kidney transplants. This indicates that the expression of *Fgl2* within the CD8^+^ T cell population does not directly contribute to the maintenance of allotransplant tolerance. Thus, our finding of elevated *Fgl2* expression results from the immune microenvironment in the accepted kidney, such as increased levels of IFN-γ, a cytokine shown to induce *Fgl2* expression (20–22). Our finding that T cells in the accepted kidney allografts increase IFN-γ secretion despite the absence of antigen stimulation corroborates prior studies that showed that IFN-γ abundance drives Tregs to restrain tumor-specific T cell function in tumor-draining lymph nodes or the tumor microenvironment (29, 30), that IFN-γ is secreted by inducible Tregs and has a crucial role in allografts tolerance (31–34), and that CD8^+^ central memory cells promote tolerance in a murine lung transplantation model through TNF-α– and IFN-γ–mediated mechanisms (35). To determine whether CD8^+^ T cells are required for the induction and maintenance of kidney allograft tolerance, we transplanted DBA/2J kidneys into B6.CD8-KO recipients. In our study, DBA/2J heart allografts were rejected in these KO mice, but the kidney allograft was accepted long term. These data demonstrate that CD8^+^ T cells are not needed for the induction and maintenance of kidney allograft tolerance in our model. Our finding that PD-1–KO recipients reject kidney allografts confirmed that T cell exhaustion is essential for kidney allograft tolerance. Furthermore, our current study revealed that reprogramming of donor-reactive T cells is mediated by IFN-γ, as shown by the adoptive transfer of donor-sensitized IFNGR-KO T cells that resulted in graft rejection. These results show that the CD8^+^ T cells possess the capacity to reject transplants, but are prevented from rejecting allografts by reprogramming that occurs within the accepted kidney to exhausted/regulatory-like cells via an IFN-γ–mediated mechanism, rendering them innocuous via a process we call “defensive tolerance.”

To assess whether accepted kidney allografts can defend against presensitized cells, we adoptively transferred donor-alloreactive CD45.1^+^ T cells into CD45.2 B6 recipients before or after the transplantation of DBA/2J kidney allografts. While transfer of the alloreactive T cells prior to renal transplantation resulted in the loss of spontaneous acceptance of the kidney, rejection was not observed if the adoptive transfer of alloreactive cells occurred after the kidney transplant had developed rTLOs. In these experiments, isolation of CD45.1^+^ T cells from an accepted kidney versus a rejecting graft showed a higher percentage of CD122^+^PD-1^+^ CD8^+^ T cells, known as regulatory-like CD8^+^ T cells (16, 36). In contrast, Eomes^+^PD-1^+^ CD8^+^ T cells, known as exhausted CD8^+^ T cells (37, 38), were more prevalent in rejecting allografts. Whether these divergent populations can be used as reliable biomarkers to assess if an allograft is undergoing the process of acceptance versus rejection is a focus of ongoing investigation.

We believe that “defensive tolerance,” which can protect normal allografts from rejection, has similarities with tolerance that are mediated by malignant tumors. In our scRNA-seq analysis of T cells from spontaneous murine tumor models, we found exhausted and regulatory-like T cells, including *Cd8*^+^*Fgl2*^+^*Il2rb^+^* cells. We hypothesize that in the models of both spontaneous acceptance of kidney allografts and spontaneous murine tumors, infiltrating proinflammatory cells are reprogrammed to innocuous cells because of the pro-tolerance microenvironment that consists of regulatory TLOs. One might further postulate that the establishment of “defensive tolerance” may contribute to the exhaustion or dysfunction that is observed in CAR-T cells when targeting solid tumors (39–41).

In summary, reprogramming of infiltrating cytotoxic CD8^+^ T cells to an exhausted/regulatory-like phenotype occurs within the accepted kidney allograft mediated by IFN-γ. A “tolerant” environment must be in place for reprogramming to occur. Adoptive transfer of alloreactive T cells before the transplantation of kidney allografts results in rejection. We believe that the reprogramming of cytotoxic CD8^+^ T cells into innocuous cells results from what we have termed “defensive tolerance.” Our further understanding of how this reprogramming is accomplished in some allografts or tumors has implications for designing more effective, clinically applicable treatment protocols for achieving allotransplant tolerance, as well as giving us an understanding of tumor immunobiology.

## Methods

### Sex as a biological variable.

Our study exclusively examined male mice. We have done some kidney allotransplants using female recipients and have obtained similar results with regards to the induction of tolerance, but there was greater variability regarding survival rates, not due to rejection of the renal allograft, but from technical complications resulting from our DBA/2J-to-B6 kidney transplantation technique when using female recipients.

### Mice.

The C57BL/6J (B6, H2^b^), DBA/2J (H2^d^), B6.129S2-*Cd8a^tm1Mak^*/J (CD8-KO), B6.129S7-*Ifng^tm1Ts^*/J (IFN-γ–KO), B6.Cg-*Pdcd1^tm1.1Shr^*/J (PD-1–KO), B6.*Ptprc*^a^ *Pepc*^b^/BoyJ (CD45.1), B6.129S7-*Ifngr1^tm1Agt^*/J (IFNGR-KO), B6.129S6(Cg)-*Ptf1a^tm2(cre/ESR1)Cvw^*/J, B6.129P2-*Trp53^tm1Brn^*/J, and B6.129S4-*Kras^tm4Tyj^*/J strains were purchased from The Jackson Laboratory. The B6.*Fgl2*^–/–^ (Fgl2-KO) mice were a gift from Mandy Ford (Emory University School of Medicine, Atlanta, Georgia, USA) and Vijay Kuchroo (Harvard Medical School and Brigham and Women’s Hospital, Boston, Massachusetts, USA). All mice were maintained under pathogen-free conditions in filter-top cages throughout the experiments with an automatic water system and were cared for according to methods approved by the American Association for the Accreditation of Laboratory Animal Care.

### Kidney transplantation.

Kidney transplantation was performed as detailed previously (28). In brief, a cuff of the aorta and inferior vena cava was anastomosed in an end-to-side manner. The ureter was anastomosed to the urinary bladder. A bilateral nephrectomy was also simultaneously performed.

### Skin transplantation.

Full-thickness skin allografts (1 cm × 1 cm) harvested from donor mice were transplanted to the recipient’s lateral flank and were held in place by sutures. A gauze dressing was maintained for 7 days after the transplantation to prevent the graft from being dislodged. Graft survival was monitored by daily visual inspection, and rejection was determined when greater than 80% of the graft became necrotic.

### Heart transplantation.

Mouse hearts were transplanted to a heterotopic abdominal location with appropriate microsurgical anastomoses as previously described (42). DBA/2J donor hearts were transplanted into C57BL/6J or B6.CD8-KO recipients. Transplanted hearts were monitored by direct transabdominal palpation at least twice a week. The vigor of contraction of the transplants was recorded on a scale of 0–3+ (43).

### Histological and immunopathological analysis.

Sagittal sections of allografts were fixed in formalin and sections were stained with hematoxylin and eosin (H&E) and periodic acid–Schiff (PAS). Immunohistochemistry was performed using anti-Foxp3 (FJK-16s, Invitrogen/Thermo Fisher Scientific, 14-5773-37), individually and as a double stain with anti-CD3 (polyclonal, DAKO Agilent, A045201-2). Other antibodies included anti-CD8 (4SM15, Invitrogen/Thermo Fisher Scientific, 14-0808-82) and anti-CD45.1 (A20, Abcam, ab25078). Pathologic evaluation was done using an Olympus BX53 microscope equipped with a digital camera (DP76, Olympus).

### Isolation of kidney and spleen cells.

Prior to tissue collection, the kidney was perfused using a collagenase solution (2 mL of 1× HBSS, 1 mL of collagenase A, and 3 μL of DNase I). The kidney tissue was harvested, manually ground down, and digested in the collagenase solution. The remaining undigested tissue was manually ground down using a 70-μm strainer, washed 3 times, and resuspended using FACS buffer. The spleen was collected in RPMI and then ground down using a syringe and 70-μm strainer. One milliliter of ACK Lysis Buffer (Gibco/Thermo Fisher Scientific) was added to remove red blood cells from the spleen. Cells were washed 3 times and resuspended in FACS buffer.

### Flow cytometry analysis.

Cells were collected at scheduled time points and stained with viability dye (eFlour 506, 1:1,000 dilution; Invitrogen, L34976 A). After viability staining, CD16/32 Fc Block (1:100 dilution; BioLegend, 101302) was added to each sample for a 5- to 10-minute preincubation step. After Fc Block, 2 staining panels were created using conjugated monoclonal antibodies. One panel consisted of anti-FGL2 (Alexa Fluor 488; Invitrogen, PA5-71472), anti–PD-1 (PE-Cy7; Biolegend, 109109), anti-CD122 (BV421; BD Biosciences, 562960), anti-CD45.1 (Percp-Cy5.5; BioLegend, 110727), and anti-CD4 (APC-Cy7; BioLegend, 100413). The other panel consisted of anti-FGL2 (Alexa Fluor 488), anti–PD-1 (PE-Cy7), anti-CD122 (BV421), anti-CD45.1 (Percp-Cy5.5), and anti-CD8a (APC-Cy7; BioLegend, 100713). The surface markers were added to the samples at a 1:200 dilution for each antibody for 30 minutes (Supplemental Table 2). Following incubation of the surface markers, anti-Foxp3 (APC; Invitrogen, 17-5773-80) and anti-Eomes (PE; BioLegend, 157705) intracellular staining was performed. The samples were resuspended in FACS buffer and analyzed via flow cytometry on a BD Biosciences FACSVerse instrument. Gating strategies were controlled using fluorescence-minus-one (FMO) controls and universal negatives (Supplemental Figure 8). For negative controls, unstained cells or cells stained with each isotype-controlled monoclonal antibody were utilized. All samples were analyzed on a FACSVerse with FlowJo software (Tree Star). Figures from flow cytometry are representative of triplicate samples.

### scRNA-seq of kidney allografts and spleen.

Isolated kidney or spleen cells were sorted for viable CD45^+^ cells via flow cytometry using an anti-CD45 antibody (BioLegend, 103114) and a fixable viability dye (Invitrogen, L34976 A). These cells were used to construct scRNA-seq libraries on a 10× Genomics Chromium instrument using a Chromium Next GEM Single Cell 3′ kit, which were sequenced in paired-end fashion (44) on an Illumina HiSeq 2500 instrument to a depth of approximately 100 million read pairs per sample.

### Bioinformatics analyses of scRNA-seq data of kidney allografts and spleen.

The raw sequencing data underwent initial mapping and processing using the CellRanger package (10× Genomics). The resulting read counts were further analyzed using Seurat (45, 46) and Monocle 3 (17–19). This included filtering cells by the number of unique molecular identifiers (UMIs), mitochondrial content, and number of expressed genes and further normalization and scaling of read counts. Data sets for various time points (1 week, 3 weeks, and 24 weeks for accepted recipient and 1 week for rejecting recipient) and biological replicates (*n* = 3 at 1 week, *n* = 5 at 3 weeks, and *n* = 3 at 24 weeks) were integrated, followed by PCA, generation of UMAP plots, and cell clustering using Seurat functions with default parameters. All mouse replicates were integrated using Seurat after scRNA-seq was performed. This creates a data object for all pooled samples together. While each mouse is a separate sample, replicates in a single time point are pooled and compared between other time points. Annotation of cell types was performed manually by investigating canonical markers in the literature. Gene expression analysis across time and cluster type was performed using Seurat software (45, 46) and Nebulosa packages (47).

Integrated data from Seurat analysis were then transformed into a Monocle 3 object, including expression data and cell-level metadata using the as.cell_data_set function to allow trajectory analysis. This object underwent further analysis, including unsupervised clustering of cells using the cluster_cells function and updating the Monocle 3 object. Next, the Monocle 3 object underwent analysis using the learn_graph function to determine the biological program of gene expression for the scenario in question and, therefore, learned the trajectory of cells through this higher-dimensional space. Lastly, the order_cells function chooses root states using the interactive Monocle 3 online software and determines the pseudotime values based on the object produced using the learn_graph function. When this was complete, various qualitative graphs were produced to illustrate the trajectories of cell types in a larger population.

### scRNA-seq data of KPC tumor.

A mouse model of pancreatic ductal adenocarcinoma was employed that combined the tamoxifen-inducible pancreatic acinar cell–specific expression of Cre recombinase (*Ptf1a*-CreER) with the expression of oncogenic Kras^G12D^ (LSL-Kras^G12D^) and heterozygous loss of *Tp53^+/fl^* (48–50). Mice were administered tamoxifen at 6 weeks of age and tumors harvested for scRNA-seq analysis at 6 months of age. Approximately 50 mg of tumor was enzymatically disassociated in RPMI containing 0.1 mg/mL DNase I (Roche), 0.2 mg/mL collagenase P (Roche), 0.1 mg/mL Dispase (Gibco/Thermo Fisher Scientific), and 2% fetal bovine serum (FBS). The cell suspension volumes were calculated for a target cell recovery of between 4,000 and 8,000 cells and loaded on the Chromium instrument using the 10× Genomics Chromium Single Cell 3′ Reagents v3 kit according to the manufacturer’s guidelines. Purified cDNAs were quantified using High Sensitivity D5000 ScreenTapes and Reagents on an Agilent Tapestation (Agilent). The final single-cell 3′ libraries were quantified using an Agilent Tapestation with High Sensitivity D1000 ScreenTapes and Reagents. Libraries were loaded at 160 pM on an Illumina cBOT and sequenced on a HiSeq 4000 for 28 base pairs on the first read, followed by an 8–base pair index read, and a 91–base pair second read, using 2 HiSeq 4000 SBS kits, 50 cycles. Illumina Real Time Analysis software was employed to generate sequence intensity files that were then demultiplexed and aligned to the human genome, version hg38, using the 10× Genomics CellRanger v3.0.1 software package.

### Bioinformatics analyses of scRNA-seq data of tumor data sets.

Tumor-infiltrating immune cells in the pancreatic cancer cell (Panc02-SIY) data sets GSM6048775, GSM6048776, GSM6048777, and GSM6048778 contained in data set GSE201026 (51), and colon cancer cell (mc38) data sets GSM5460383, GSM5460384, GSM5460385, and GSM5460386 contained in data set GSE180296 (52), were downloaded from the NCBI Gene Expression Omnibus (GEO). Cells from tumor data sets were included with the following exclusion criteria: (a) cells with greater than 10% mitochondrial genes (b), cells expressing fewer than 200 genes or more than 7,000 genes, and (c) cells with fewer than 1,000 UMIs or greater than 40,000 UMIs, and further normalization and scaling of read counts. Data sets were individually integrated, followed by PCA, generation of UMAP plots, and cell clustering using Seurat functions with default parameters. Annotation of cell types was performed manually by investigating canonical markers in the literature. Gene expression analysis in T cell subsets was performed using Seurat software (45, 46), Nebulosa packages (47), and Monocle 3 packages (17–19).

### Adoptive cell transfer.

To isolate identifiable donor-sensitized cells, DBA/2J skin grafts were transplanted into B6.*Ptprc*^a^ *Pepc*^b^/BoyJ (CD45.1) or B6.129S7-*Ifngr1^tm1Agt^*/J (IFNGR-KO) mice. Donor-sensitized recipient spleen cells were isolated 30 days after the skin allograft was rejected. The cells were sorted using a pan-T cell isolation kit (Miltenyi Biotec) according to the manufacturer’s protocol. Sorted cells were resuspended in RPMI and then injected intravenously into WT CD45.2 B6 recipient or B6.*Ptprc*^a^ *Pepc*^b^/BoyJ (CD45.1) through the tail vein. Cells (2 × 10^6^) were injected approximately 2 months after kidney transplantation or in untreated recipient mice 3 days prior to the kidney transplantation. Serum BUN and creatinine levels were monitored before and after the adoptive cell transfer of alloreactive T cells to ascertain kidney function. Cells were recovered from kidney allograft, spleen, and mesenteric lymph nodes 14 days after adoptive transfer or when mice were euthanized due to kidney allograft rejection and analyzed by flow cytometry. Pathological examination was performed on kidney allograft samples.

### ELISPOT.

IFN-γ–producing cells were quantified using the mouse IFN-γ ELISpot kit (R&D Systems) according to the manufacturer’s instructions. The cells were sorted using a Pan T Cell Isolation Kit (Miltenyi Biotec) or CD8^+^ T Cell Isolation Kit (Miltenyi Biotec) according to the manufacturer’s protocol. A total of 5 × 10^5^ T cells or CD8^+^ T cells enriched from spleen cell suspensions of the donor-sensitized mouse or kidney allografts and recipient spleen in kidney-transplanted recipients were placed in each antibody-precoated well and were cocultured with intact donor (DBA/2J) and self (B6) splenocytes at 37°C in 5% CO_2_. After 48 hours of incubation, biotinylated anti–IFN-γ antibodies were added overnight and then detected with streptavidin-conjugated alkaline phosphatase and 5-bromo-4-chloro-3′-indolylphosphate *p*-toluidine salt/nitro-blue tetrazolium chloride (BCIP/NBT) substrate. Resulting spots representing IFN-γ–producing responders were counted and analyzed by ImmunoSpot Analyzer (Cellular Technology Limited).

### Suppression assay.

Isolated cells from accepted kidney allografts at 3 weeks after transplantation were sorted for viable CD8^+^CD122^+^ or CD8^+^CD122^–^ T cells via flow cytometry using an anti-CD8 antibody (APC-CY7), anti-CD122 antibody (BV421), and a fixable viability dye (eFlour 506, 1:1,000 dilution; Invitrogen, L34976 A). Splenic CD4^+^CD25^–^ responder cells purified from naive mice were labeled using the CellTrace Violet kit (Thermo Fisher Scientific). The cells were cocultured with CD8^+^CD122^+^ or CD8^+^CD122^–^ T cells from accepted kidney allografts or splenic CD4^+^CD25^+^ Tregs from naive mice with or without anti-CD3/anti-CD28 beads (Dynabeads, Gibco) for 72 hours. Cells were retrieved and analyzed by flow cytometry. Labeled cells with dilute fluorescence were considered proliferating cells.

### Statistics.

Data are presented as mean ± standard deviation (SD) for technical replicates and mean ± SEM for biological replicates. Variables among groups were compared using the 2-tailed Student’s *t* test for comparison of 2 conditions and 1-way ANOVA test for comparison of more than 2 conditions. A *P* value of less than 0.05 was considered significant. Allograft survival curves were constructed by the Kaplan-Meier method, and comparisons were performed using the log-rank test. These analyses were performed with Prism v10.0 (GraphPad Software) and SPSS Statistics v28.0 (IBM).

### Study approval.

All research with animal models was subject to prior review and approval and conducted in compliance with institutional guidelines set forth by the Animal Care and Use Committees of the Massachusetts General Hospital.

### Data availability.

Raw and processed scRNA-seq data have been deposited in the NCBI GEO database (GSE252337) .All other raw data are provided in the Supporting Data Values file. Other data are available upon reasonable request from the corresponding authors, subject to institutional review and approval.

## Author contributions

TY and AA conceptualized the study. TY, ESS, IR, MTG, ASL, TB, DAR, MP, PSR, JCM, RBC, and AA developed the methodology. TY, ESS, IR, MTG, ASL, TB, DAR, MP, JA, PSR, JCM, RBC, and AA conducted experiments. TY, ESS, IR, MTG, ASL, TB, DAR, MP, and AA analyzed the data. TY, ESS, and AA wrote the original draft of the manuscript, which was reviewed and edited by TY, ESS, IR, MTG, ASL, TB, DAR, MP, JA, ABC, PSR, JCM, RBC, and AA. TY, JCM, RBC, and AA acquired funding. AA supervised the study.

## Supplementary Material

Supplemental data

Supporting data values

## Version 1 06/18/2024

In-Press Preview

## Version 2 08/15/2024

Electronic publication
