# Supplementary material for: Dysfunction of infiltrating cytotoxic CD8+ T cells within the graft promotes murine kidney allotransplant tolerance
Source: J Clin Invest. 2024 Jun 18;134(16):e179709. doi: 10.1172/JCI179709 (PMC11324304; doi:10.1172/JCI179709)
Supplement: Supplemental data [file jci-134-179709-s257.pdf]

Supplementary Material

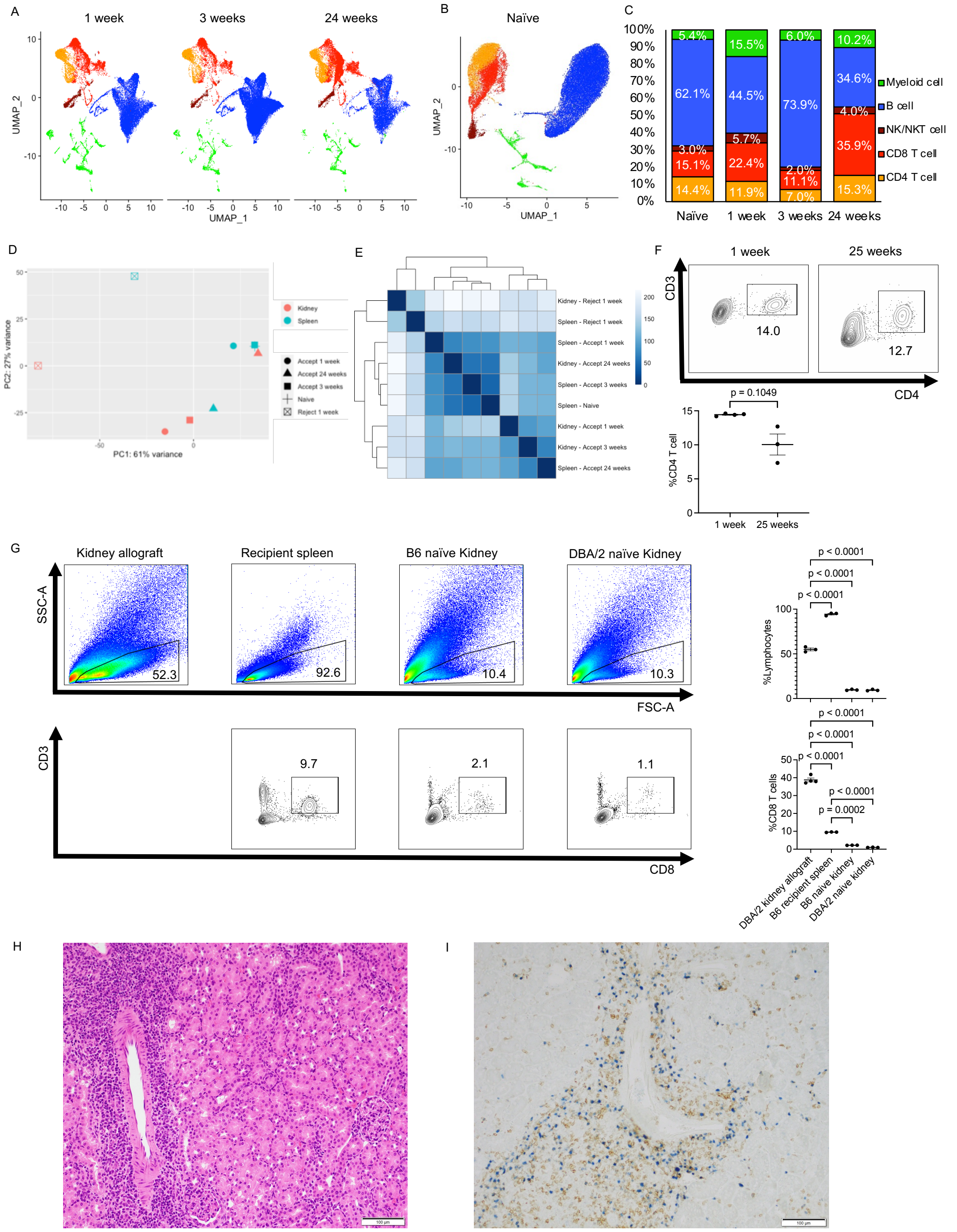

Supplemental Figure 1. Single-cell RNA sequencing and flow cytometric analysis of immune cell populations. (**A**, **B**) UMAP plot of immune cell populations in scRNAseq data of recipient spleen at each time point (**A**) and naïve spleen (**B**). (**C**) Bar graph of immune cell population in recipient spleen and naïve spleen. (**D**, **E**) PCA (**D**) and heatmap (**E**) of sample-to-sample distance analysis in total immune cells in kidney and spleen from accepted, rejecting, and naïve mice. (**F**) Frequency of $CD4^{+}$ T cells in viable lymphocytes obtained from accepted kidney allografts at one week and 25 weeks post-transplant. Data are represented as mean ± SEM, compared by 2-tailed Student's t-test. (**G**) Frequency of lymphocytes per total cells (top) and $CD8^{+}$ T cells in viable lymphocytes (bottom) in accepted kidney allografts and recipient's spleen at 1-week post-transplant, and B6 and DBA/2 naive kidney were analyzed. Data are represented as mean ± SEM, compared by 1-way ANOVA test. (**H, I**) Histopathological findings of H&E staining (**H**) and immunohistochemistry of CD3 (brown) and Foxp3 (blue) (**I**) in accepted kidney allografts at 2 weeks. Low magnification with the scale bar indicated 100μm.

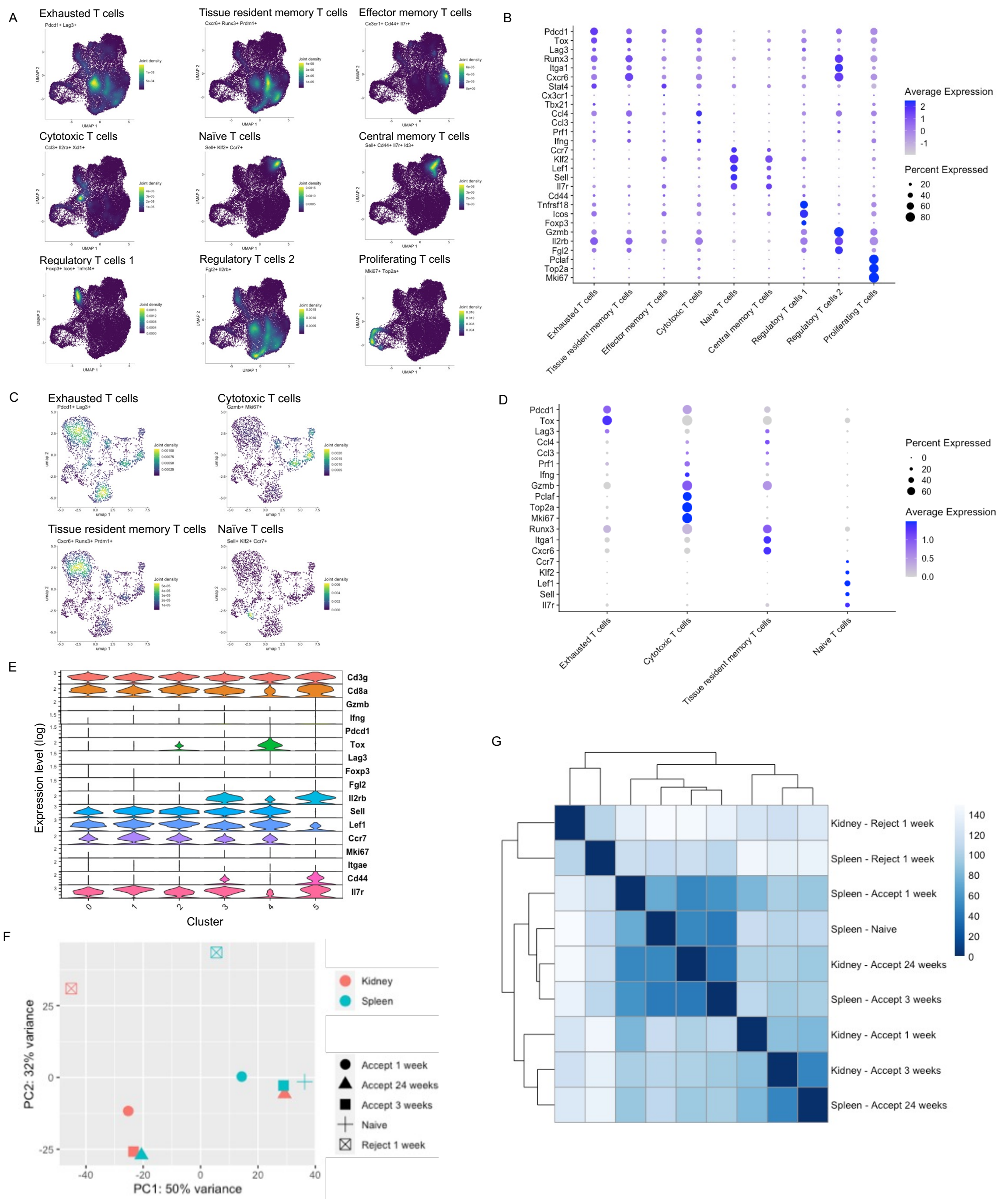

Supplemental Figure 2. scRNAseq analysis in CD8$^+$ T cell subset. (**A**–**D**) Joint density plots identify CD8$^+$ T cell subpopulations based on multiple gene expressions (**A**, **C**) and dot plot of gene expression in CD8$^+$ T cell cluster (**B**, **D**) in accepted (**A**, **B**) and rejecting kidney allografts (**C**, **D**). (**E**) Violin plots of cytotoxic, exhaustion/regulatory, naïve, and memory cell marker genes in CD8$^+$ T cell subsets in naive spleen. **F**, **G** PCA (**F**) and heatmap (**G**) of sample-to-sample distance analysis in CD8$^+$ T cells in kidney and spleen from accepted, rejecting, and naïve mice.

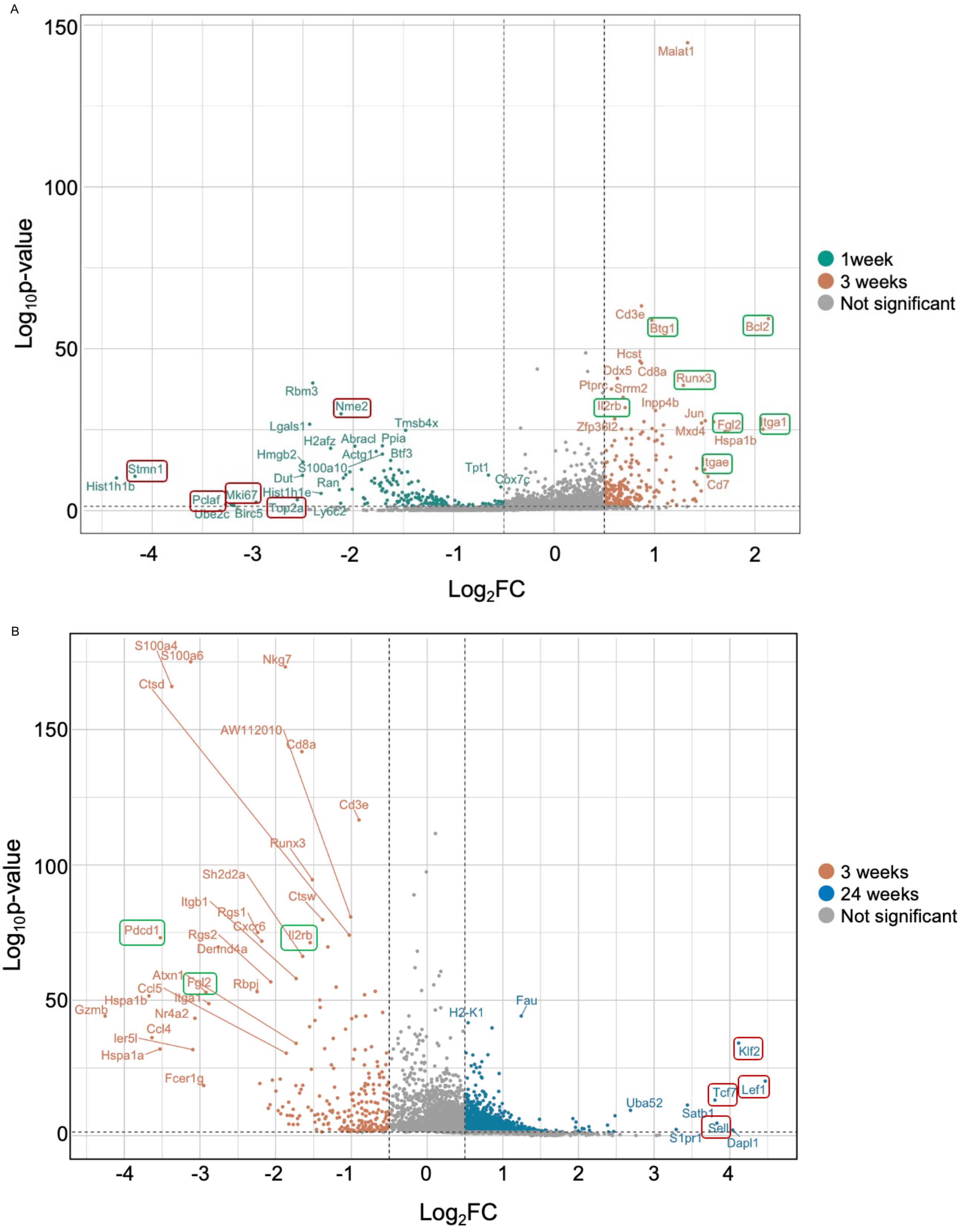

Supplemental Figure. 3. Volcano plots of differentially expressed gene analysis in CD8⁺ T cells cluster in accepted kidney allografts. (**A**, **B**) Volcano plots of comparison of differential gene expression analysis in CD8⁺ T cell clusters between 1 week and 3 weeks (**A**) and 3 weeks and 6 months (**B**).

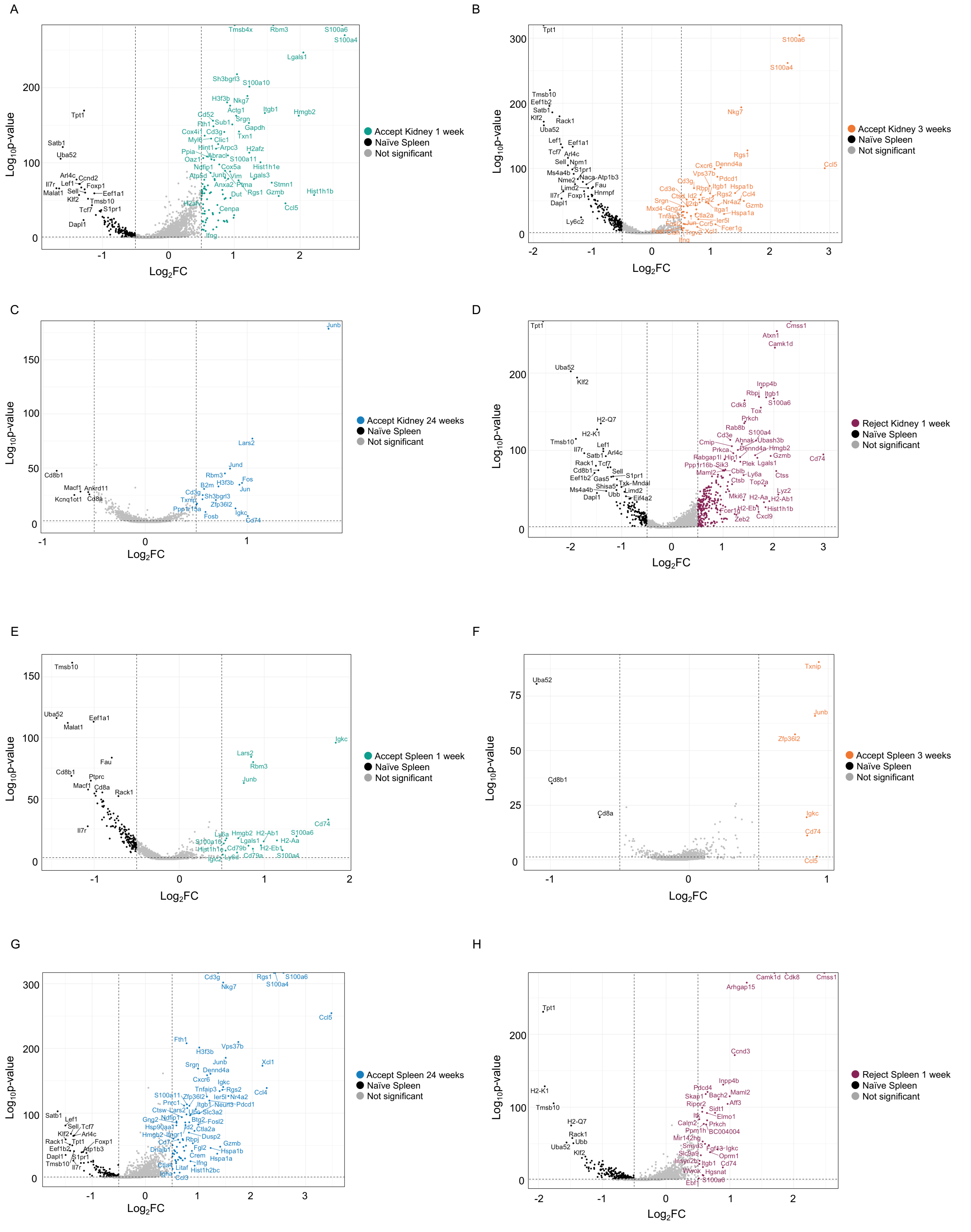

Supplemental Figure. 4. Volcano plots of differentially expressed gene analysis in CD8$^+$ T cells in each sample compared with naïve spleen. (**A**–**C**) Accepted kidney allografts at 1 week (**A**), 3 weeks (**B**), 24 weeks (**C**). (**D**) Rejecting kidney allografts at 1 week. (**E**–**G**) Accepted recipient spleen at 1 week (**E**), 3 weeks (**F**), 24 weeks (**G**). (**H**) Rejecting recipient spleen at 1 week.

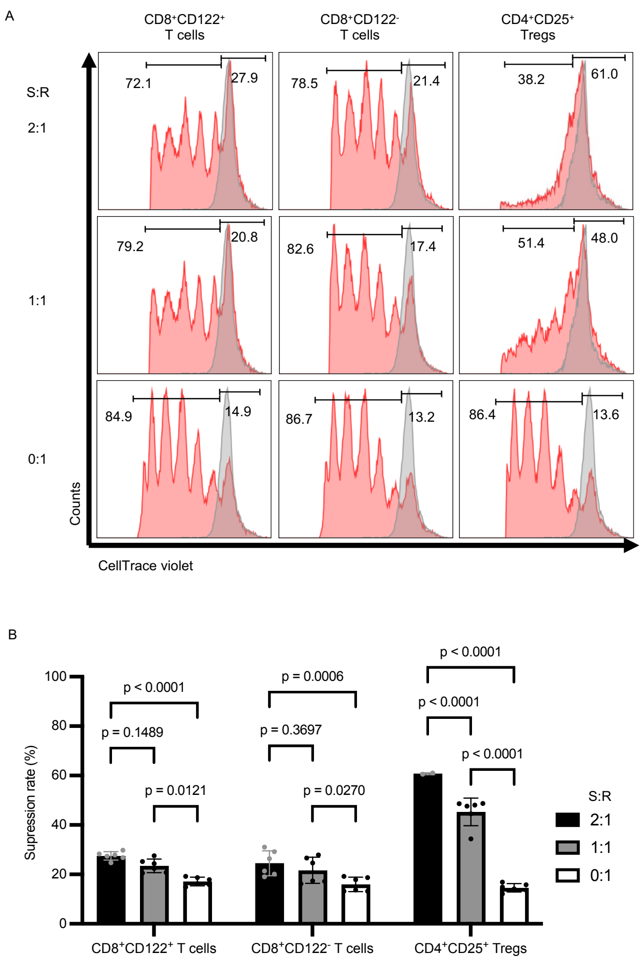

Supplemental Figure 5. Suppression assay. (**A**) Suppressor cells are $CD8^+CD122^+$ T cells and $CD8^+CD122^-$ T cells obtained from accepted kidney allografts and $CD4^+CD25^+$ Tregs from naive spleen. Suppressor: Responder ratios (S:R) are 2:1 (upper row), 1:1 (middle row), and 0:1 (bottom row). (**B**) Suppression rates were compared for each S:R ratio within each suppressor cell group. Data are represented as mean ± SD, compared by 2-way ANOVA test.

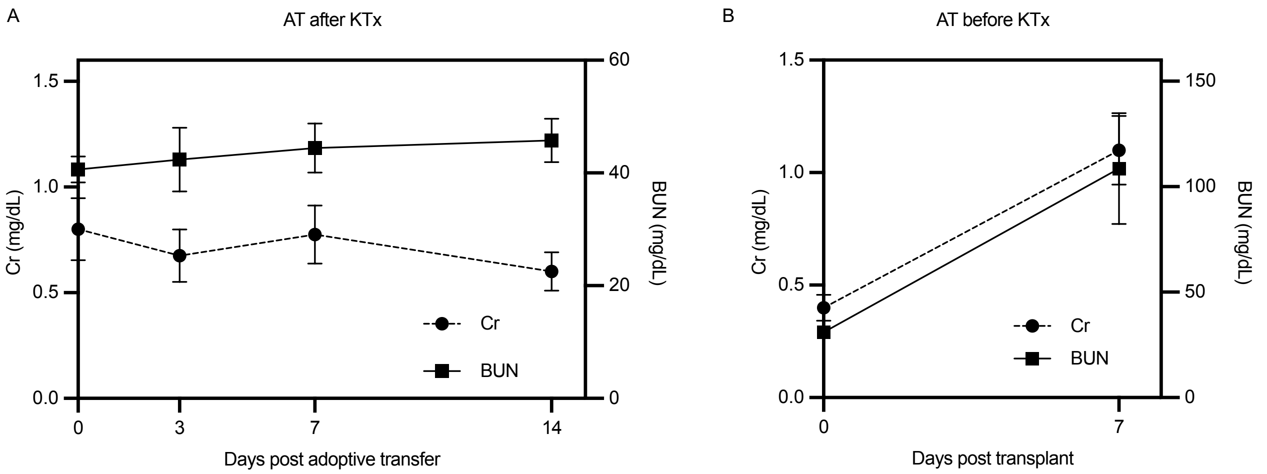

Supplemental Figure 6. Line graph of serum creatinine levels (Cr) and blood urea nitrogen (BUN). (**A**) adoptive transfer (AT) after kidney transplantation (KTx) recipients (n=4). (**B**) AT before KTx whose grafts were rejected (n=3). Data represent the mean ± SEM.

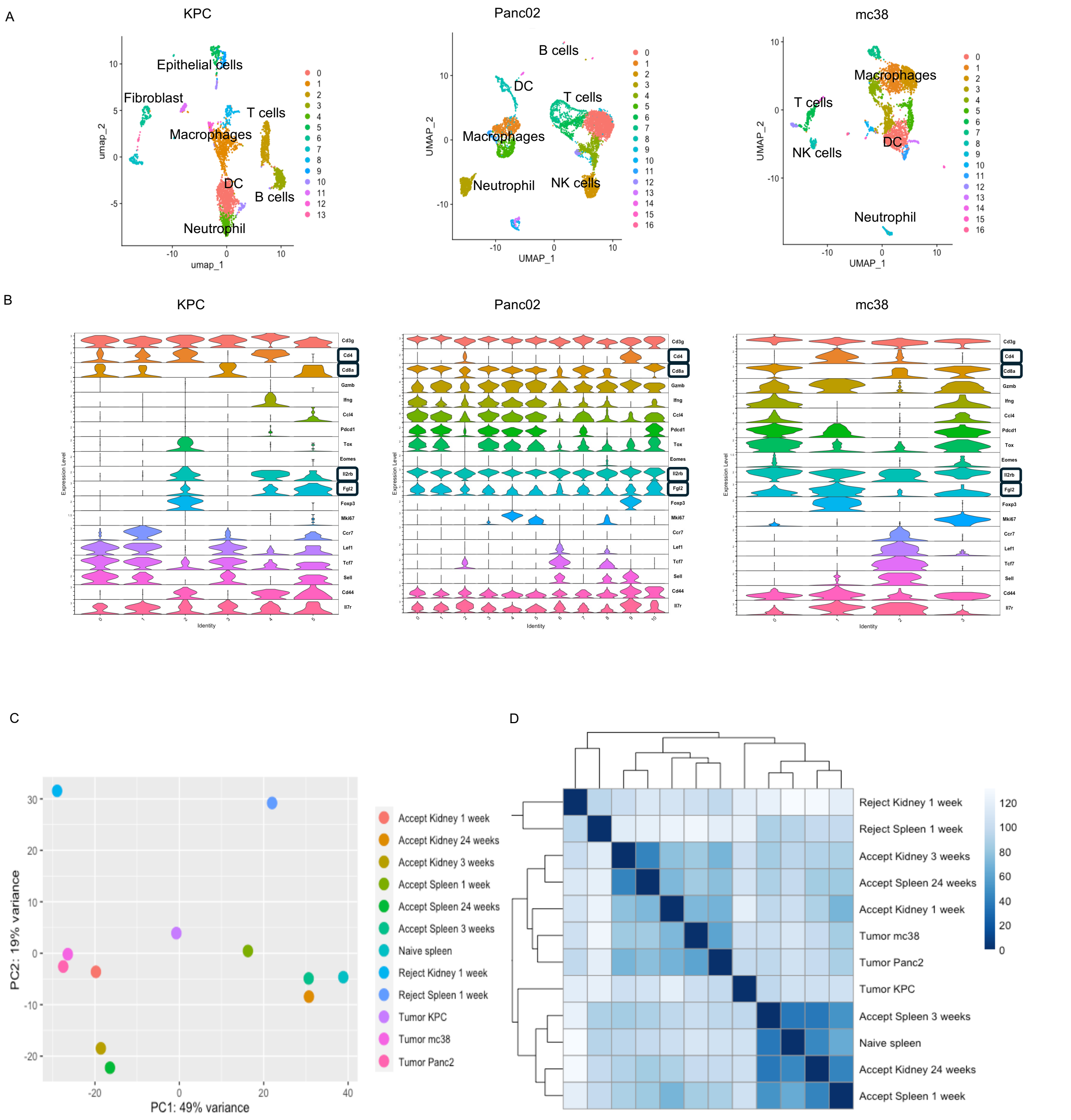

Supplemental Figure 7. scRNAseq data analysis of pancreatic and colorectal tumors in mice. (**A**) UMAP plots of total cells in KPC tumor (left), Panc02 tumor (middle), and mc38 tumor (right) infiltrated immune cells. (**B**) Violin plot of cytotoxic, exhausted, regulatory, naïve, memory T cell marker gene expression in T cell clusters in KPC tumor (left), Panc02 tumor (middle), and mc38 tumor (right). (**C**, **D**) PCA (**C**) and heatmap (**D**) of sample-to-sample distance analysis in CD8^{+} T cells in kidney and spleen from accepted, rejecting, and naïve mice and T cells in pancreatic and colorectal tumors in mice. Pancreatic cancer cell (Panc02-SIY) data sets, GSM6048775, GSM6048776, GSM6048777, and GSM6048778, contained in dataset GSE201026, and colon cancer cell (mc38) data sets, GSM5460383, GSM5460384, GSM5460385, and GSM5460386, contained in dataset GSE180296 were downloaded from the Gene Expression Omnibus. DC: dendritic cells.

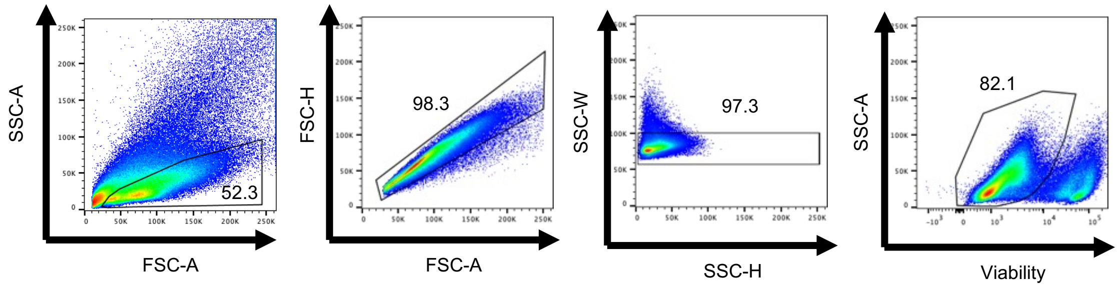

Supplemental Figure 8. Gating strategy. Gating strategy for flow cytometry.

Supplemental Table 1. Major CD8 T cell population based on gene expression.

		Accept			Reject
	**Naïve**	**1 week**	**3 weeks**	**24 weeks**	**1 week**
**Kidney**	-	CTL, Tex, Proliferative	TRM, Tex, Treg, CTL	Naive, Tcm	CTL, Tex, Proliferative
**Spleen**	Naïve, Tcm	Naïve, Tcm	Naïve, Tcm	CTL, Tex, Treg, TRM	Naïve, Tcm

CTL: cytotoxic T lymphocyte, Tex: exhausted T cell, TRM: tissue resident memory T cell, Treg: Regulatory T cell, Tcm: central memory T cell

Supplemental Table 2. Antibody information

Antibody	Color	Manufacturer	Catalog #	Clone	Dilution	Isotype
**anti-mouse PD1**	PE-Cy7	Biolegend	109109	RMP1-30	1:400	Rat IgG2b, k
**anti-mouse CD4**	APC-Cy7	Biolegend	100413	GK1.5	1:800	Rat IgG2b, k
**anti-mouse Cd16/32**	Unconjugated	Biolegend	101302	93	1:100	
**anti-FGL2**	Unconjugated	Invitrogen	PA5-71472	Polyclonal	1:200	Rabbit, IgG
**anti-mouse Eomes**	PE	Biolegend	157705	W17001A	1:400	Rat IgG2b, l
**anti-mouse CD8a**	APC-Cy7	Biolegend	100713	53-6.7	1:800	Rat IgG2a, k
**anti-mouse CD122**	BV421	BD Biosciences	562960	TM-B 1	1:400	Rat IgG2b, k
**anti-mouse CD45.1**	Percp-Cy5.5	Biolegend	110727	A20	1:800	Mouse (A.SW) IgG2a, k
**anti-mouse Foxp3**	APC	Invitrogen	17-5773-80	FJK-16s	1:400	Rat IgG2a, k
**anti-mouse CD8a**	FITC	Biolegend	100706	53-6.7	1:800	Rat IgG2a, k
**anti-mouse CD3e**	Percp-Cy5.5	Biolegend	100217	17A2	1:800	Rat IgG2b, κ
**Viability Dye**	eFluor 506	Biolegend	103137	30-F11	1:1000	--
**Zip Alexa Fluor 488 reactive dye**	Alexa Fluor 488	Invitrogen	Z11233	--	1:200	--
**CellTrace™ Violet Cell Proliferation Kit**	Violet (405/450)	Invitrogen	C34557	--	1:1000	--
